# Alternative conformations of a group 4 Late Embryogenesis Abundant protein associated to its in vitro protective activity

**DOI:** 10.1038/s41598-024-53295-7

**Published:** 2024-02-02

**Authors:** David F. Rendón-Luna, Inti A. Arroyo-Mosso, Haydee De Luna-Valenciano, Francisco Campos, Lorenzo Segovia, Gloria Saab-Rincón, Cesar L. Cuevas-Velazquez, José Luis Reyes, Alejandra A. Covarrubias

**Affiliations:** 1 Departamento de Biología Molecular de Plantas, Instituto de Biotecnología, Universidad Nacional Autónoma de México, Avenida Universidad 2001, Colonia Chamilpa, 62210 Cuernavaca, Morelos México; 2 Departamento de Ingeniería Celular y Biocatálisis, Instituto de Biotecnología, Universidad Nacional Autónoma de México, Avenida Universidad 2001, Colonia Chamilpa, 62210 Cuernavaca, Morelos México; 3 Departamento de Bioquímica, Facultad de Química, Universidad Nacional Autónoma de México, Ciudad Universitaria, 04510 Ciudad de México, México; 4 Present Address: Programa de Biología Sintética, Centro de Ciencias Genómicas, Universidad Nacional Autónoma de México, Av. Universidad 2001, Colonia Chamilpa, 62210 Cuernavaca, Morelos México

**Keywords:** Chaperones, Drought

## Abstract

Late Embryogenesis Abundant (LEA) proteins are a group of intrinsically disordered proteins implicated in plant responses to water deficit. In vitro studies revealed that LEA proteins protect reporter enzymes from inactivation during low water availability. Group 4 LEA proteins constitute a conserved protein family, displaying in vitro protective capabilities. Under water deficiency or macromolecular crowding, the N-terminal of these proteins adopts an alpha-helix conformation. This region has been identified as responsible for the protein in vitro protective activity. This study investigates whether the attainment of alpha-helix conformation and/or particular amino acid residues are required for the in vitro protective activity. The LEA4-5 protein from *Arabidopsis thaliana* was used to generate mutant proteins. The mutations altered conserved residues, deleted specific conserved regions, or introduced prolines to hinder alpha-helix formation. The results indicate that conserved residues are not essential for LEA4-5 protective function. Interestingly, the C-terminal region was found to contribute to this function. Moreover, alpha-helix conformation is necessary for the protective activity only when the C-terminal region is deleted. Overall, LEA4-5 shows the ability to adopt alternative functional conformations under the tested conditions. These findings shed light on the in vitro mechanisms by which LEA proteins protect against water deficit stress.

## Introduction

Due to their sessile nature, plants must withstand harsh environmental conditions during different developmental stages. Abiotic stresses such as drought, heat, cold, salinity and flooding, among others, cause adverse impacts to the growth and reproduction of different plant species, including crops. To cope with these stressful environments, plants have developed a wide diversity of strategies. These adaptive and adjustment responses modify different processes at physiological, metabolic, cellular, and genetic levels^1^. At the genetic level, the expression of an assortment of genes is altered through diverse molecular mechanisms, promoting their induction or repression, leading to changes in the amount of their corresponding products. Among the genes whose expression is induced in response to environmental stress, particularly conditions involving water deficit, are those encoding the so-called Late Embryogenesis Abundant (LEA) proteins. These proteins present high abundance during the late stage of embryogenesis, at the onset of the seed desiccation process, characteristic that led to their name^2,3^.

Most LEA proteins have distinctive physicochemical properties: as hydrophilins, in general, they are small and hydrophilic; they present a biased composition enriched in small and charged residues, and with low content of cysteines, aromatic and hydrophobic amino acids. These features qualify them as Intrinsically Disordered Proteins (IDPs)^4–6^. The presence of unique conserved motifs in the different plant LEA proteins led to their classification in at least seven groups^4,7^. Alternative classifications based on the conserved motif principle exist. Pfam employs a similar criterion but assign distinct nomenclature. Some categorizations focus exclusively on Arabidopsis LEA proteins^8,9^, while others consider shorter conserved motifs^10^. Noteworthy, LEA proteins have been identified in all plant species where they have been searched^7^.

Remarkably, LEA-like proteins have been found in organisms different from plants, particularly, those able to withstand desiccation conditions, an ability known as anhydrobiosis^11–13^. In vitro data suggest that LEA proteins have protective functions, preventing the denaturation and consequent aggregation of other proteins when exposed to low water availability, imposed by partial or total dehydration or freeze–thaw treatments^14,15^. Other stressful conditions such as oxidative environments or extreme temperatures have been tested^16–18^. Also, in vitro experiments allowed to propose that they may act as membrane stabilizers and as metal scavengers^12,19,20^.

In this work, we focused on group 4 LEA (Pfam LEA_1) proteins, a highly conserved family in plants, characterized by a conserved N-terminal region, and a C-terminal region, variable in sequence and length. In different plant species, this family is composed by few members. The *A. thaliana* genome contains three genes for LEA4 proteins in chromosomes 1 (*LEA4-1*), 2 (*LEA4-2*), and 5 (*LEA4-5*)^8,9^. Mutations by T-DNA insertion which resulted in a knockdown mutant in *AtLEA4-5* gene showed reduced germination and fewer reproductive structures under water limitation^21^, demonstrating the involvement of these proteins in the plant response to these stressful conditions. A chaperone-like mechanism of action for this set of proteins has been proposed based on in vitro protection assays, where two of these proteins (AtLEA4-2 and AtLEA4-5) prevent the inactivation of lactate dehydrogenase (LDH), used as a reporter enzyme, subjected to partial dehydration or to freeze–thaw treatments^22^*.* Further analysis of this activity showed that the AtLEA4-5 N-terminal region but not the C-terminal one retains the protective activity. Moreover, it was shown that the N-terminal region acquires alpha helicity under high osmolarity, by increasing glycerol concentrations, or under high macromolecular crowding induced by the addition of polyethylene glycol (PEG). This effect was not detected for the C-terminal region^22^. These results pointed to a possible action mechanism, where the AtLEA4-5 protective activity mostly depends on its N-terminal region, involving the conserved motifs and or its ability to gain alpha-helix as central elements for this function.

To investigate the contribution of these elements to the in vitro AtLEA4-5 protective activity, in this work we generate different mutants affecting the conserved motifs and amino acid residues, or the ability to gain helicity to evaluate their effect on their protective activity. The results here show that the ability to gain alpha-helix structure of the AtLEA4-5 protein plays a significant role in the in vitro chaperone-like function only when the N-terminal region is separated from the C-terminal region; however, the reduction of the helicity did not alter the protecting effect when the C-terminal region is present. These data indicate that, while the C-terminal region does not have a protector capacity by itself, it contributes to the in vitro protective function of the AtLEA4-5 protein. Moreover, this finding reveals the possibility that AtLEA4-5 protein exhibits more than one functional conformation, one where the protein gains helicity and other where this capacity is lost, and where inter-chain electrostatic interactions may play a central role in its protective function. These findings shed light on the intricate mechanisms by which LEA proteins protect against water deficit stress.

## Results

### The LEA4 proteins of land plants cluster in two subgroups

**Figure 1:**
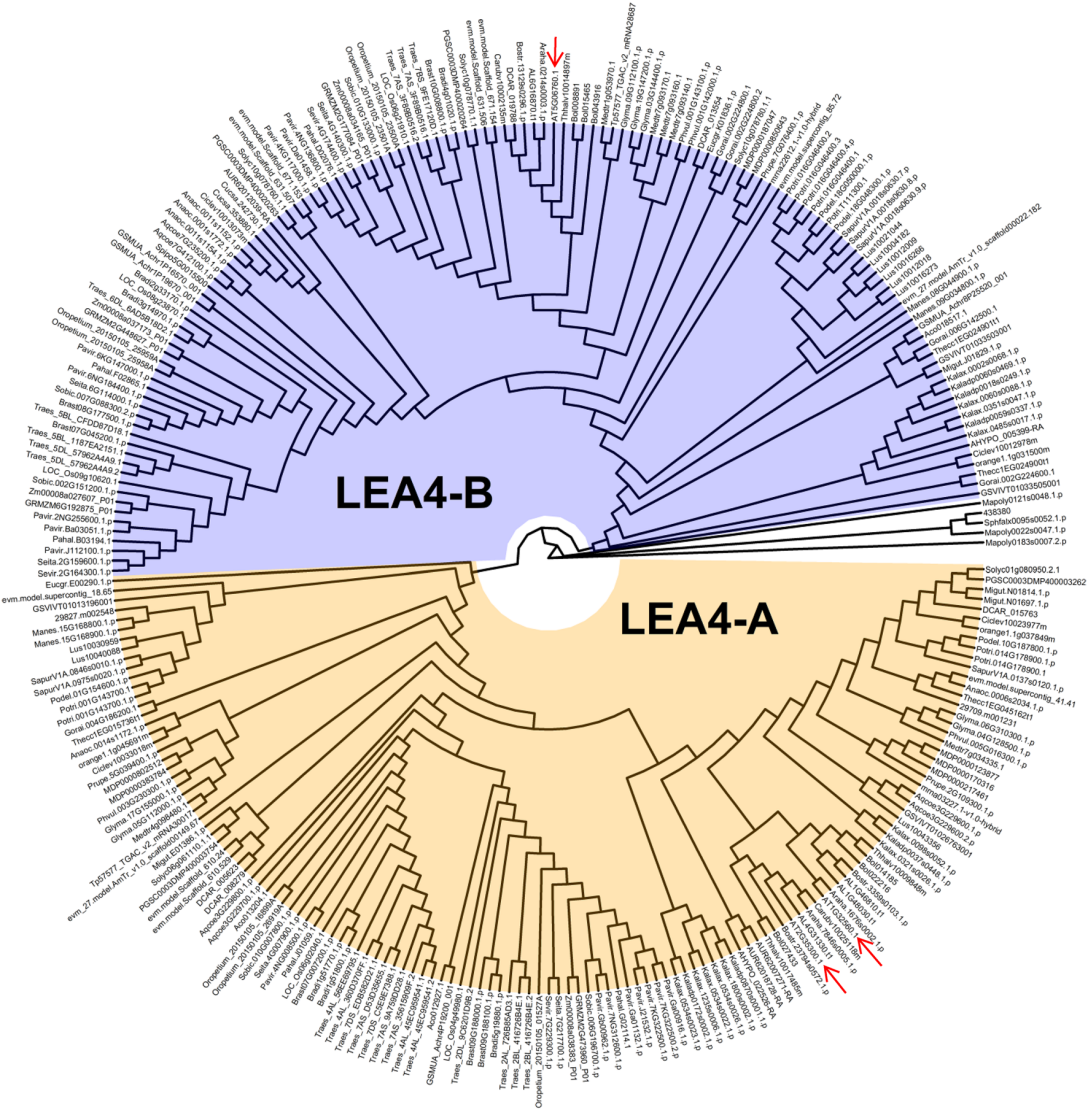
Phylogenetic analysis of group 4 LEA proteins in plants. Schematic phylogenetic relationship between plant LEA4 proteins showing the clustering into two subgroups (LEA4-A and LEA4-B). LEA4 related proteins were also found in seedless species, exhibiting their ancestral origin (non-color branches). The Phytozome protein codes are shown at the end of each branch. Arabidopsis LEA4 proteins are highlighted with arrows.

**Figure 2:**
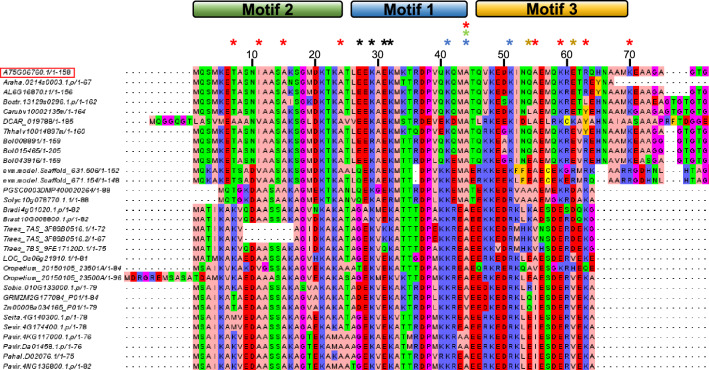
Multiple sequence alignment (MSA) of the N-terminal region of 30 representative LEA4 proteins showing the closest phylogenetical relation to AtLEA4-5 protein. Alignment visualization was performed by Jalview software. Residues were colored as follows: red: negatively charged residues; blue: positively charged residues; green: polar uncharged residues; pink; residues with hydrophobic side chain; magenta: glycine and proline; orange: tyrosine and phenylalanine, and yellow: cysteine. Colored asterisks represent the location of the amino acid residues modified in the mutants generated from AtLEA4-5. Black: LEA4-5-NT1; blue: LEA4-5-NT2; green: LEA4-5-NT3; yellow: LEA4-5-NT4; red: LEA4-5-NT9P. Motifs 1, 2 and 3 were considered according to Battaglia et al.^4^. The numbers in the upper part of the figure correspond to the AtLEA4-5 protein sequence. LEA4-5 protein from Arabidopsis is highlighted in a red box.

To extend the phylogenetic analysis of LEA4 family and look in more detail on its origin and diversification, we performed a BLASTp search using the AtLEA4-5 protein sequence as query on the NCBI non-redundant database. The BLASTp detected that the N-terminal region of the protein aligned with a consensus sequence designated as LEA_1 superfamily (consensus domain PFAM03760). We mined the Phytozome database (version 11) with the detected consensus LEA_1 PFAM. We cleaned the selected sequences to make a multiple-sequence alignment (MSA) to carry out a phylogenetic analysis of the group 4 LEA proteins. Redundant sequences were identified and removed through clustering, while inaccurate annotations were systematically eliminated during the cleaning process. We obtained 275 protein sequences annotated as LEA4 proteins. After the phylogenic analysis, we identify 136 proteins that belong to LEA4 subgroup A and 134 to the LEA4 subgroup B, while 5 corresponded to hypothetical LEA4 proteins from basal plants. These results agree with previous reports showing the initial ancient duplication of LEA4 family, and the diversification of these proteins early in their evolution into two subgroups^4^. Furthermore, by including the annotated LEA4 proteins from bryophytes (*Marchantia polymorpha* and *Sphagnum fallax*) and from a spike moss (*Selaginella moellendorffii*), we found that this protein family may have emerged in the origin of land plants; however, they do not group within any of the LEA4 subgroups, indicating that the emergence of the two divergent groups possibly came with the appearance of seed plants (spermatophytes) (Fig. 1). The MSA showed the expected conservation of their N-terminal regions, which mostly contains the so-called LEA_1 protein family consensus sequence as defined by the Pfam database. This analysis also showed that the most conserved motif in LEA_1 Pfam domain is Motif 1, followed by motifs 2 and 3 (Fig. 2 and Supplementary Fig. S4). Noteworthy features include the conserved enrichment of charged residues in motifs 1 and 3, the high percentage of serine residues in motif 2, and the abundance of glycine and proline residues in the C-terminal regions of sub-group B proteins. The tyrosine residues located around the end of the C-terminal region also showed high conservation. The residues showing the utmost conservation in all analyzed sequences were Ala24 and Ala44, which are surrounded by highly conserved serine and threonine residues. A proline residue was also highly conserved in the N-terminal region of LEA4 proteins in sub-group B (Fig. 2 and Supplementary Fig. S4). The analysis of the isoelectric point (pI) of all LEA4 proteins considered in this study revealed that most of them present a pI, above 9; yet there are few LEA4 proteins showing an acidic pI (Supplementary Fig. S5). Arabidopsis LEA 4 proteins present basic pIs, 8.95 for AtLEA4-1, 9.67 for AtLEA4-2, and 9.22 for AtLEA4-5. The basic nature of the LEA4 family points out to the involvement of the positive charged residues in the selective interactions that these proteins may establish with their targets.

### The most conserved amino acid residues in the AtLEA4-5 N-terminal region are not required for its in vitro protective activity

**Figure 3:**
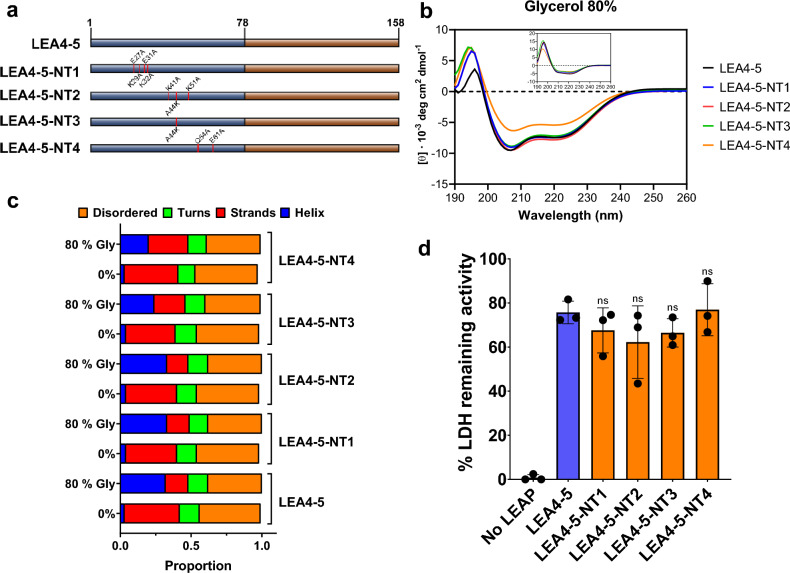
AtLEA4-5 mutants where the most LEA4 protein conserved residues were changed. (**a**) Schematic localization of the point mutations in each AtLEA4-5 mutant. (**b**) CD spectra of mutant proteins in 80% glycerol solution. Inset shows the corresponding differential spectra. [Φ] = Molar ellipticity. (**c**) Dichroweb analysis showing the fractions of different secondary structures in wild-type and mutant proteins. (**d**) Protecting activity of wild-type and mutant proteins in freeze–thaw assays using LDH as reporter enzyme. These data was obtained from three independent experiments with three technical replicates, using 10:1 LEA:LDH molar ratio. Error bars represent the standard deviations between samples. *ns* non statistically difference as compared to LEA4-5.

In this work, we focused on the functional analysis of AtLEA4-5 protein, as representative of LEA4 protein family. To get insight into the AtLEA4-5 function, we selected those amino acid residues showing the highest conservation in the N-terminal region, which was shown to be sufficient for AtLEA4-5 in vitro protective activity^22^. Residues E27, K29, E31, K32, K41, A44, K51, Q54, and E61 were replaced with others with contrasting physicochemical properties, as follows: E27A, K29A, E31A, K32A, K41A, A44K, K51A, Q54A, and E61A (Fig. 2, Supplementary Fig. S4). Most of the mutations were to alanine residues, an amino acid lacking a sidechain past the β-carbon that does not introduce conformational flexibility^23^. These mutations were distributed in four different AtLEA4-5 mutants, LEA4-5-NT1 to NT4 (Fig. 3a). As the AtLEA4-5 N-terminal region is predicted in silico to form alpha-helix conformation, and it was demonstrated that it acquires helicity under high osmolarity and macromolecular crowding conditions^22^, we evaluated the predicted propensity to form alpha-helix of the wild type and the different AtLEA4-5 mutants in silico. The results did not show considerable differences between the propensity towards alpha-helix formation and disorder tendency between wild-type and mutant proteins (Supplementary Figs. S6 and S7), in consonance with the CD spectra obtained for each purified protein (Supplementary Fig. S2A) in solution without glycerol or with 40% and 80% glycerol (Fig. 3b and c, Supplementary Fig. S8, Supplementary Table S4). This was evident from their differential spectra obtained by subtracting the data from 0% glycerol to the data obtained from 80% glycerol (Fig. 3b inset). As it could be expected, these data demonstrate that the modifications introduced in this set of AtLEA4-5 mutant proteins do not compromise their capacity to gain alpha-helix structure.

The impact of these mutations in the AtLEA4-5 protective function was evaluated by freeze–thaw treatments, where the availability of water is reduced, affecting the activity of the reporter enzyme^14,22^. The results from these assays did not show difference in the protective activity of these mutants as compared to the wild-type protein (Fig. 3d), indicating that changes in the most conserved residues of LEA4-5 do not lead to detrimental effects on its protective capacity under the conditions tested.

### The carboxy-terminal region contributes to the AtLEA4-5 protective effect

**Figure 4:**
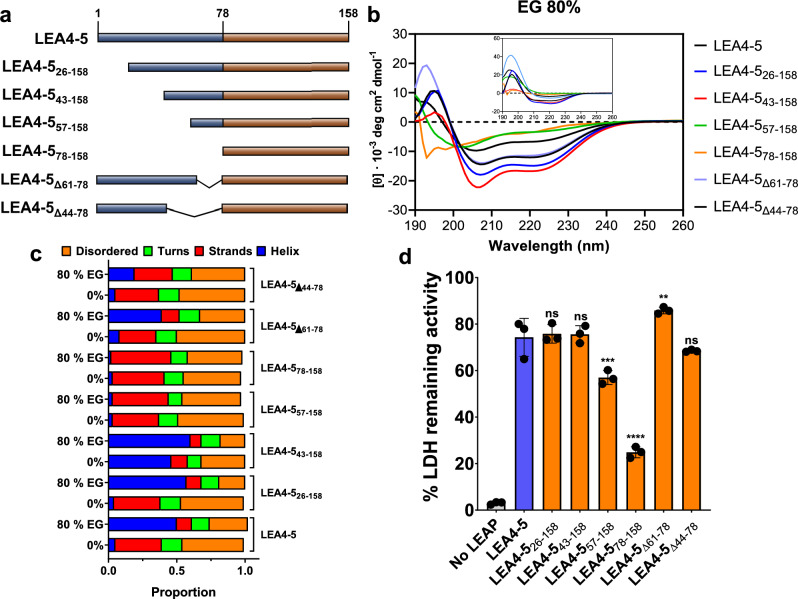
AtLEA4-5 deletion mutants. (**a**) Schematic description of the deletions generated from AtLEA4-5 protein. (**b**) CD spectra of the deletion mutant proteins in 80% ethylene glycol (EG) solution. Inset shows the corresponding differential spectra. [Φ] = Molar ellipticity. (**c**) Dichroweb analysis showing the fractions of different secondary structures in wild-type and mutant proteins. (**d**) Protecting activity of wild-type and mutant proteins in freeze–thaw assays using LDH as reporter enzyme. These data come from three independent experiments with three technical replicates, using 10:1 LEA:LDH molar ratio. Error bars represent the standard deviations between samples. *ns* non statistically difference as compared to LEA4-5. **p < 0.01, ***p < 0.001, ****p < 0.0001.

To further search for those AtLEA4-5 regions that contribute to preserve the activity of target proteins under freeze–thaw stress, we generated deletion mutants LEA4-5_26–158_, LEA4-5 _43–158_, and LEA4-5_57–158_, where protein segments of different sizes were removed from its N-terminal (25, 42, and 56 residues, respectively) (Fig. 4a, Supplementary Figs. S1 and S2B). LEA4-5_26–158_ lacks only the region where an amphipathic helix was predicted^22^, while in LEA4-5_43–158_ and LEA4-5_57–158_, the second and third regions, respectively, with predicted alpha-helix were further eliminated (Supplementary Figs. S6 and S7). Consistent with the secondary structure propensity predicted, data obtained from their CD spectra using EG shows that the protein LEA4-5_57–158_ in which most of the helical propensity region was removed, lost its ability to form helices under high osmolarity. Dichroweb analysis indicated that the region between 43 and 57 residues mostly contributes to the gain of alpha-helix in AtLEA4-5 protein (Fig. 4b and c, Supplementary Fig. S8, Supplementary Table S4). EG was used instead of glycerol to make the CD experiments easier to handle because EG has a lower viscosity. We assured that the same osmotic potential as that provided by the glycerol concentrations formerly was used. Concerning the protective activity of these variants, we observed no discernible difference in the protective activity of the LEA4-5_26–158_ and LEA4-5_43–158_ truncated mutants compared to the LEA4-5 protein (Fig. 4d). However, when 56 residues from the N-terminus were removed to generate LEA4-5_57–158_, a significant decrease in its protection activity was obtained (58%) (Fig. 4d), which was further reduced when 77 residues were deleted to obtain LEA4-5_78–158_, leaving the so-called C-terminal region (24%) (Fig. 4d and Supplementary Fig. S3a–c). LEA4-5_78–158_ mutant lost the propensity to form alpha-helix (Supplementary Figs. S6 and S7), and the ability to gain helicity under high EG concentrations as shown by the CD experimental data (Fig. 4b and Supplementary Fig. S8), consistent with its high level of structural disorder. Yet, this mutant still showed 24% of protection as compared to 74% detected for the wild-type protein, implying that it also contributes to the protective activity of the full-length LEA4-5 protein (Fig. 4d).

**Figure 5:**
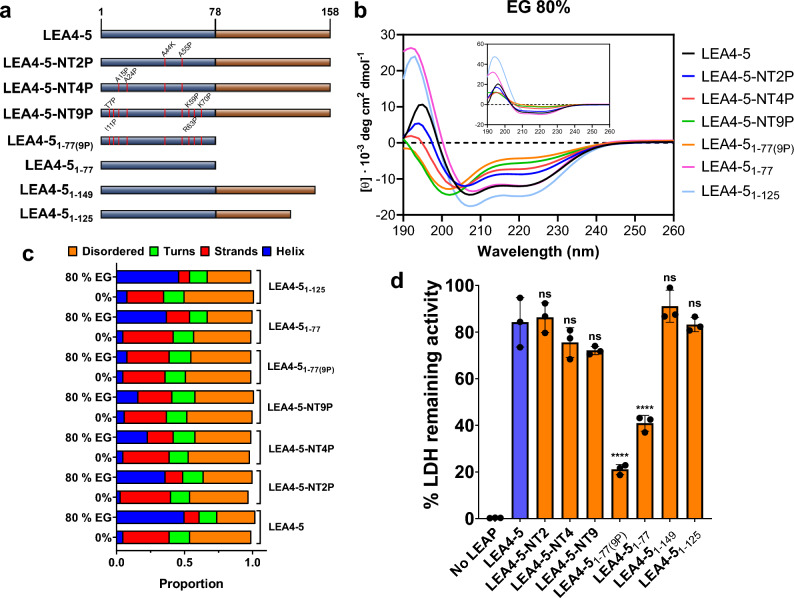
Impact of proline insertions in the N-terminal region and the complete AtLEA4-5 protein on their secondary structure and protective activity. (**a**) Schematic description of AtLEA4-5 mutants containing proline residues, and of two deletion mutants at the C-terminal end. (**b**) CD spectra of mutant proteins in 80% ethylene glycol (EG) solution. Inset shows the corresponding differential spectra. [Φ] = Molar ellipticity. (**c**) Dichroweb analysis showing the fractions of different secondary structures in wild-type and mutant proteins. (**d**) Protecting activity of wild-type and mutant proteins in freeze–thaw assays using LDH as reporter enzyme. These data come from three independent experiments with three technical replicates, using 10:1 LEA:LDH molar ratio. Error bars represent the standard deviations between samples. *ns* non statistically difference as compared to LEA4-5. ***p < 0.001, ****p < 0.0001.

Analysis of the C-terminal region shows the presence of two tyrosine residues at the end, which could contribute to its protective function by establishing intra- or inter-hydrophobic or electrostatic interactions. To investigate the tyrosine possible role on this function, two deletion mutants removing the regions comprising one or both tyrosine residues were constructed (LEA4-5_1–149_ and LEA4-5_1–125_) (Fig. 5a); however, the lack of these regions did not cause any detrimental effect on their protective activity under the conditions tested (Fig. 5d).

Because the LEA4-5_57–158_ deletion mutant showed a reduced protective activity as compared to the wild-type protein, the possibility that the region between 44 and 78 residues could contribute to this activity was addressed by the construction of two additional deletion mutants, LEA4-5_Δ61-78_ and LEA4-5_Δ44-78_, which lack the tracts between 61–78 and between 44–78 residues, respectively (Fig. 4a and Supplementary Fig. S1). The results from the CD analyses showed that the increase in helicity in the presence of EG (80%) for the LEA4-5_Δ61-78_ variant was 39%, i.e., 11% lower than the wild-type protein (0.5), whereas LEA4-5_Δ44-78_ achieved an even lower fraction of helicity (0.19) (Fig. 4b and c, Supplementary Fig. S8 and Table S4). These results indicate that the regions removed in these mutants are required for the AtLEA4-5 acquisition of alpha helix conformation under these EG treatments. However, the freeze–thaw protection assays showed that both deletion mutants achieved a protective activity like the wild-type protein (Fig. 4d), suggesting that the removed residues do not directly contribute to the AtLEA4-5 protecting effect, and that the remaining sequence and-or the attained helicity of these LEA4-5 variants under low water availability are sufficient to provide protection under stress.

### The capacity to gain helicity of the AtLEA4-5 N-terminal region under high osmolarity is required for its optimal protective activity

**Figure 6:**
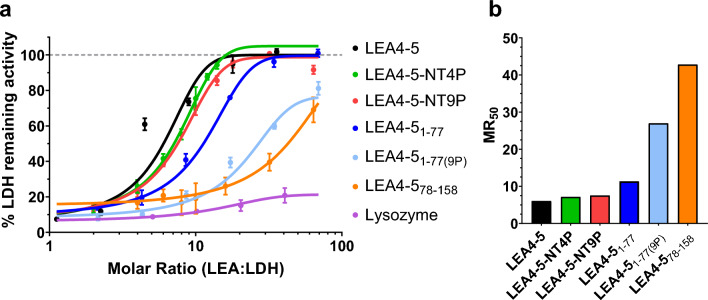
Protecting activity upon freeze–thaw treatments at different molar ratios of wild-type and mutants of LEA4-5. (**a**) These data come from three independent experiments with three technical replicates, using the molar ratio (LEA:LDH) indicated in the graph. Error bars represent the standard deviations from the mean. The x-axis values are in log_10_ scale. (**b**) Estimated molar ratio needed to attain 50% protection activity (MR_50_).

In previous studies, Cuevas-Velazquez, et al.^22^ found that the LEA4-5 N-terminal region (LEA4-5_1–77_) gains high helicity proportion under high osmolarity coincident with high protection levels for proteins under stress. Based on these results, it was proposed that the formation of the alpha-helix could be relevant for the LEA4-5 protective function. To test this hypothesis, we generated a mutant, where the helicity in LEA4-5_1–77_ was disrupted by replacing nine amino acid residues by proline residues at positions interrupting regions with high propensity to form alpha-helix (T7P, I11P, A15P, A24P, A44P, A55P, K59P, R63P, K70P) (LEA4-5_1–77(9P)_) (Fig. 5A and Supplementary Fig. S1). The introduction of the nine proline residues decreased the mean hydropathy and net charge of the resultant protein (Table S5) and, as expected, reduced the propensity of this polypeptide to form alpha-helix (Supplementary Figs. S6 and S7). This effect was verified by CD using protein solutions without or with addition of EG (40% and 80%) (Fig. 5b and c, Supplementary Fig. S8) showing a lower alpha-helix acquisition at 80% EG for LEA4-5_1–77(9P)_, as compared to the wild-type LEA4-5_1–77_ (Fig. 5c and Table S4). Interestingly, freeze–thaw protection assays showed that the introduction of proline residues, leading to a reduction in the gain of alpha-helix under high osmolarity, negatively impacted the protective activity of this mutant protein. Of note, in this work we found that the LEA4-5_1–77_ protection effect was reproducibly lower, under different molar ratios, than that obtained by the wild-type protein (Figs. 5d and 6). In summary, these data support the need for an alpha-helix conformation in this region to provide protection to the target enzyme upon freeze–thaw treatment and indicated that both AtLEA4-5 N- and C-terminal sections contribute to AtLEA4-5 protective activity. It could be inferred that the region in LEA4-5_1–77_ has more influence in this activity than that in LEA4-5_77–158_, given its higher protection effect as compared to that conferred by the C-terminal region (Fig. 6a and b).

### The requirement of helicity for AtLEA4-5 protective activity is not evident in the presence of its C-terminal region in the complete protein

Because our data indicated that both regions of the AtLEA4-5 protein contribute to its optimal protection activity, mutants containing two (LEA4-5-NT2P), four (LEA4-5-NT4P) or nine (LEA4-5-NT9P) proline residues in the N-terminal region were now generated in the context of the complete LEA4-5 protein (Fig. 5, Supplementary Fig. S1). The CD data showed as expected that the ability to gain helicity under high osmolarity conditions decreases with increasing the number of proline residues, yet LEA4-5-NT9P retained a substantial alpha-helix fraction of 0.16 in 80% EG, which is even higher than that for LEA4_1-77(9P)_ corresponding to 0.08 (Fig. 5b and c, Supplementary Fig. S8, Supplementary Table S4). Intriguingly, when the protective activity of these mutants (LEA4-5-NT2P, LEA4-5-NT4P, and LEA4-5-NT9P) was determined in the freeze–thaw assays, all of them exhibited similar levels of activity to that achieved by the wild-type protein (Figs. 5d and 6a and b). These data may imply that, in the proline-mutants, the presence of the unstructured carboxy-terminal region (residues 78–158) promotes conformational changes that allow the proper protein structure for recovery of the AtLEA4-5 in vitro protective activity.

## Discussion

The physicochemical properties and the high structural flexibility of some IDPs represent a challenge for the elucidation of their mechanisms of action. Numerous efforts have been made to understand the function of LEA proteins, a set of plant IDPs involved in the response to stressful environments, representing model systems to study the molecular activity of IDPs under adverse conditions. In this work, by combining bioinformatic, structural, biochemical, and functional in vitro analyses of wild type and mutant proteins, we deepen into the mechanism of the protective function of a group 4 LEA protein. LEA4 proteins constitute a highly conserved family in the plant kingdom, phylogenetically grouped in two subgroups (A and B), where the N-terminal region shows the highest conservation, whereas the C-terminal region varies in length showing low conservation among plant species^22^. LEA4 proteins in subgroup B accumulate in vegetative tissues of various plant species subjected to water-limiting conditions and are highly abundant in orthodox dry seeds^7^. The analyses of LEA4 protein conformational properties in different environments showed that the structural landscape of these IDPs changes with a reduction in water availability and an increase in macromolecular crowding, showing a gain of helicity and loss of disorder. These data suggested that the sequence conservation and the alpha-helix acquisition, most probably formed in the N-terminal region of these proteins, have a central role in their in vitro protective function^22^.

In this work, by the functional and structural characterization of different mutants of AtLEA4-5 protein, a member of the subgroup B LEA4 proteins, we found that even though its N-terminal region plays a dominant role for its in vitro protective activity on the LDH reporter enzyme during freeze–thaw treatments, the C-terminal region is also required for an optimal protection. Freeze–thaw treatments lead to the denaturation and aggregation of LDH, among other enzymes^24^ and they have been used to monitor the protective activity of different LEA proteins on different labile enzymes to this treatment. The association between LDH and some LEA proteins has been reported suggesting a chaperone-like mode of action^25–27^. Despite the evident limitations of this and other in vitro assays to ascertain the in vivo mechanism(s) of action of LEA proteins, these have offered an experimental alternative to get some knowledge on their potential functions and the relation to their structural properties.

The results here showed that the most evolutionary conserved amino acid residues in this protein do not play a major role in its in vitro protective activity on LDH, nor in the folding of this protein under water-limiting conditions. Likewise, the removal of the first 42 amino acid residues did not show an effect on these properties. However, the elimination of additional 14 residues (LEA4-5_57–158_), containing part of the most conserved motif (Fig. 2), did affect the protein capacity to gain helicity and its protective action, indicating an involvement of the residues in this region in the AtLEA4-5 structural and functional properties. Nevertheless, the elimination of just the fragment from 44 to 78 residues in the mutants LEA4-5_Δ61-78_ and LEA4-5_Δ44-78_ did not affect their protective activity, supporting the relevance of the remaining protein regions. The deletion that eliminates the 77 residues conforming the N-terminal region, leaving only the C-terminal segment of this protein (LEA4-5_78–158_), led to the lowest protective activity among the evaluated mutant variants and to the loss of the helicity acquisition competence. The protection provided by this mutant (LEA4-5_78–158_) although low is indicative of its requirement. Therefore, these data suggested that the LEA4-5 N- and the C-terminal regions are necessary for an optimal protection activity in these in vitro assays. This requirement may involve electrostatic interactions among the amino acid residues within these protein sections, facilitating the acquisition of a functional conformation under the tested conditions (Supplementary Fig. S9A–D).

The ability of AtLEA4-5 to gain alpha-helix conformation in response to environmental conditions, the high propensity of its N-terminal region to acquire alpha helicity, and the protecting activity of this region predicted a functional relevance for this structural feature. To address the significance of this conformation, we generated mutants where the formation of alpha-helix structure was hindered. The insertion of proline residues in the N-terminal region suggests that this conformational change is needed for an optimal protection activity of this particular segment under the condition tested. Nevertheless, when identical proline insertions were introduced in the same region of the complete AtLEA4-5 (LEA4-5-NT9P), this mutant protein showed a protective function similar to that of the wild-type protein. Because the loss of helicity in the N-terminal region significantly affects its functional performance, as shown by the evaluation of LEA4-5_1–77(9P)_ mutant, this result was unexpected. The protective activity conferred by the LEA4-5-NT9P mutant protein suggests that the helicity required for an optimal protective activity of the AtLEA4-5 N-terminal region is a dispensable condition for this activity at least in the context of the complete protein, pointing out to the involvement of the AtLEA4-5 C-terminal region to attain the conformation needed for an optimal activity.

Cuevas-Velazquez et al.^28^ showed by FRET experiments that AtLEA4-5 acquires a more compact conformation under high osmolarity and high macromolecular crowding, indicating that this conformational compaction is necessary for its activity. Although in the wild-type protein, this conformation is achieved by the gain of alpha-helix, the changes introduced in the LEA4-5-NT9P mutant open the possibility that the higher structural disorder produced by the loss of helicity enables the establishment of the proper or compensatory electrostatic interactions among the oppositely charged amino acid residues positioned in the N- and C-terminal regions of this protein to generate a functional conformation under the imposed stress treatment^29^ (Supplementary Fig. S9A–D). According to this model, the functionally relevant conformation of the LEA4-5-NT9P protein may involve intrachain electrostatic attractions with the charged residues in the C-terminal region. Alternatively, it suggests that this mutant protein may adopt an extended conformation, allowing the protective effect to occur through interchain electrostatic interactions with its binding partner, LDH in this case. The functional persistence resilience of LEA4-5-NT9P, circumvent the need for alpha-helices formation, exhibiting the capability conferred by the IDP conformational malleability to establish alternative active conformations. Overall, the results in this work show that the first 43 and the last 32 amino acid residues of the AtLEA4-5 protein are not required for its protecting function, indicating that the remaining sequence have the physicochemical properties necessary to yield an optimal protection activity under the conditions imposed by the freeze–thaw treatments. Furthermore, we provide evidence indicating that the alpha-helix conformation as exhibited by the wild-type protein is not needed for its protective activity as long as the C-terminal region is present. Why then the N-terminal of LEA4 proteins shows such high conservation during evolution? The simplest answers are that the reporter enzyme LDH used in the in vitro assays is not a natural client and that the experimental treatments might be far from the actual conditions where these proteins are needed. Despite these limitations, these analyses allowed to evaluate the potential functionality of these proteins and the relation between their in vitro activity and some structural properties. One possible explanation for the conservation of a sequence conferring the ability to gain helicity of the N-terminal region of the AtLEA4-5 natural protein under particular conditions is the need for these proteins to perceive some physicochemical characteristics in the cellular environment that will be transmitted through conformational changes to the rest of the protein to get the corresponding functional structure(s). The in vitro assays do not allow us to evaluate this ability at the same time as the protective activity. Even so, our data strongly suggest that the protein unable to gain helicity but with an effective protective activity (LEA4-5-NT9P) exhibits an extended conformation, which most probably will not be able to sense the environmental changes. Further analyses, such as extending the conformational characterization of the mutant proteins using FRET, and generating and characterizing additional mutants, among others, are in progress. These studies will provide a more detailed understanding of the functional conformations associated to the in vitro function of intrinsically disordered LEA proteins and will add insights to their role in plant stress responses.

## Methods

### Phylogenetic analysis

We retrieved LEA4 protein sequences annotated with the PFAM ID LEA_1 (PF03760)^30^, an identifier of LEA4 proteins, from the Phytozome database^31^ [https://phytozome.jgi.doe.gov/pz/portal.html]. We applied the phmmer algorithm from the HMMER web server^32^ using all the sequences obtained previously to include the homologous sequences from gymnosperms. Redundancy was reduced with CD-HIT^33^, removing all sequences with a higher than 95% similarity. The resulting clustered sequences were degaped, purged, and aligned with MUSCLE software v3.8.31^34^. The resulting alignment was analyzed with ModelTest Next Generation software v.0.1.3^35^ to choose the evolution model that best fitted our data according to the Bayesian and Akaike information criteria. Once the evolution model was determined, the phylogenetic analysis was run using 1000 random seed trees; then, the substitution matrix of the evolution model was calculated with the RAxML software v.8.2.10^36^ using 1000 random seed trees. The substitution matrix of the evolution model was then obtained by applying a maximum likelihood approach. To support the topology of the tree, we performed a rapid bootstrap analysis. Two hundred resamples were used and the support value was annotated directly in the tree, along with the division between LEA4 subgroups and the taxonomic family of plants, using the “ggtree” R package, version 1.10.5^37^.

### Secondary structure and disorder prediction

The protein sequences were analyzed using FELLS^38^ to predict the protein regions that are prone to form alpha-helix, and Metapredict v2.2^39^ to calculate disordered regions according to multiple individual predictors.

### Design and generation of mutant proteins

To identify the conserved amino acid residues, we visualized the multiple sequence alignment (MSA) of the LEA4 protein sequences using Aliview^40^. We selected candidate residues for mutation considering their degree of conservation and their physicochemical properties. We evaluated the impact of each mutation using the FELLS and Metapredict algorithms. We designed a total of seventeen mutant sequences of the AtLEA4-5 protein (Supplementary Fig. S1).

The point mutations in LEA4-5-NT1 to NT4 were generated by overlap extension PCR^41^ with the designed primers for such purposes (Supplementary Table S1), using the oligonucleotide combinations described in Supplementary Table S2. We used as DNA template the AtLEA4-5 ORF, where methionine 33 was changed to leucine (LEA4-5_M33L_) to disrupt a second translation initiation site used by bacteria, avoiding the production of two LEA4-5 ORF derived proteins. Because this variant has been considered as the wild type AtLEA4-5 version, in this work we will refer to it as AtLEA4-5. A restriction site was also introduced into this template to facilitate subsequent insertions into the pTrc99A vector. The resulting PCR amplicons were cloned into the pJET1.2/blunt cloning vector using the CloneJET PCR Cloning Kit (Thermo Fisher Scientific). The cloning reaction was used to transform *E. coli* DH5α. Recovered colonies were used to obtain plasmid DNA using the GeneJET Plasmid Miniprep Kit (Thermo Fisher Scientific). All constructs were verified by DNA sequencing using the T7 forward primer. Oligonucleotides synthesis and DNA sequencing services were provided by Oligonucleotide Synthesis and DNA Sequencing Facilities of the Instituto de Biotecnología-UNAM.

AtLEA4-5 truncated mutants (LEA4-5_26–158_, LEA4-5_43–158_, LEA4-5_57–158_, LEA4-5_78–158_, LEA4-5_1–125_, LEA4-5_1–149_, LEA4-5_1–77_) were obtained by PCR using AtLEA4-5 ORF with the specific primers (Supplementary Tables S1–S3). LEA4-5_61–78_, LEA4-5_44–78_ and LEA4-5-NT2P were obtained by overlap PCR, using AtLEA4-5 ORF as template and specific primers. For LEA4-5-NT4P manufacturing, the same approach was followed but, in this case, the LEA4-5-NT2P was used as template, while for LEA4-5-NT9P, the template was LEA4-5-NT4P. LEA4-5_1–77(9P)_ was obtained by amplifying the N-terminal region of the LEA4-5-NT9P mutant. All mutants were subcloned into pJET1.2/blunt and verified by sequencing as previously described.

Recombinant proteins were expressed in *E. coli* using the pTrc99A vector. For this, the pJET1.2 derived plasmids were digested with *Sal*I and *Nco*I to release the DNA fragment of interest. The purified fragments and the digested pTrc99A vector were ligated using T4 DNA ligase (Thermo Fisher Scientific), to further transform *E. coli* DH5α. Colonies containing the desired plasmids were selected, and all generated plasmids were verified by sequencing with the pTRC99a_R primer (Supplementary Table S1).

### Protein expression and purification

Proteins were purified following the protocol described by Campos, et al.^42^ and Romero-Perez, et al.^43^ with some modifications. Briefly, purified pTrc99A derived plasmids were introduced into *E. coli* XL1-Blue. LB medium (100 mL) with ampicillin was inoculated with a single colony, incubated overnight at 37ºC, and used to inoculate fresh LB medium (1 L). After two hours, IPTG was added to a 1 mM final concentration, and the culture was pelleted after 2 h. The pellet was kept at -20°C until further processing. The pellet was thaw at room temperature, resuspended in 15 mL of Extraction Buffer (20 mM Tris pH 8.0, 10 mM NaCl), placed in a boiling water bath for 10 min, and cooled down on ice for 10 min. After centrifugation at 12,100 RCF at 4 °C for 10 min, the recovered supernatant was mixed thoroughly with concentrated trichloroacetic acid (TCA) (2% final concentration) and incubated on ice for 10 min. Then, the suspension was pelleted again, and concentrated TCA was added to 8% final concentration to the recovered supernatant, mixed thoroughly, and incubated on ice for 10 min. After one more centrifugation step, the pellet was saved and washed twice with cold acetone (− 20 °C), removing the remaining acetone by pipetting, and by evaporation in a fume hood. Finally, the pellet was resuspended in 100–500 µL of the buffer of selection. The protein solution was dialyzed against three liters of distilled water, three times, at 4 °C, using a dialysis membrane with a MWCO of 8 kDa. The purity and integrity of all protein samples were verified by SDS-PAGE and Coomassie blue staining (Supplementary Fig. S2A and B).

### Protein purification using the IMPACT-NC system

Due to the instability of the unstructured AtLEA4-5 C-terminal region (LEA4-5_78–158_), and of the LEA4-5_1–77(9P)_ mutant, they were purified using the IMPACT-NC system (New England Biolabs), in which the proteins of interest were fused to an intein for a subsequent purification through affinity chromatography using a chitin matrix. For this, LEA4-5_78–158_ and LEA4-5_1–77(9P)_, were inserted into the pTYB11 vector (New England Biolabs), verified by sequencing, and used to transform *E. coli* XL1-Blue cells. Fresh LB media without antibiotic (1 L) was inoculated with an ON culture (100 mL) obtained from one isolated colony. After incubation for 2 h at 37 °C, protein expression was induced with 1 mM IPTG for 3 h, and cells were collected by centrifugation. The procedure continued according to manufacturer’s instructions. Because LEA4-5_78–158_ protein mutant cannot be detected by Coomassie staining, the fractions of interest were identified by Western blotting, using the anti-LEA4-5-C_1 antibody (Agrisera AS22 4831). The appropriate fractions were pooled and dialyzed against Milli-Q water using 3.5 kDa MWCO membranes. The purity and integrity of LEA4-5_1–77(9P),_ protein was verified by SDS-PAGE and Coomassie blue staining (Supplementary Fig. S2), whereas for LEA4-5_78–158_, its integrity was confirmed by Western blot, using a specific antibody, and its purity by Ponceau staining because this polypeptide is not stained by Coomassie blue. This staining method showed the absence of additional polypeptides in the sample, supporting its purity (Supplementary Fig. S3A and B).

### Western blot assays

Western blots were performed following standard protocols using 15 µL of the collected fractions. LEA4-5 carboxy-terminal region antibody (Agrisera AS22 4831) was used in 1:1000 dilution, whereas secondary antibody (anti-rabbit horseradish peroxidase; Zymed) was diluted 1:10,000. Signals were developed with peroxidase substrate (Supersignal West Pico, Thermo Fisher Scientific), exposed to blue X-ray films (Kodak), and documented using ImageQuant 300 (GE Healthcare) imager.

### Protein quantification

Purified protein was quantified using the Qubit Protein Assay Kit in a Qubit 4 Fluorometer (Thermo Fisher Scientific), which has shown higher accuracy to determine the concentration of intrinsically disordered proteins^44^. The use of this quantification method was relevant to improve the quantification of protein concentrations, particularly of those proteins lacking aromatic amino acid residues (e.g. LEA4-5_1–77_).

### Circular dichroism (CD) spectroscopy

Wild type and mutant recombinant proteins were diluted to 0.3 mg/mL in 10 mM phosphate buffer at pH 8, in the absence or presence of increasing amounts of glycerol or ethylene glycol (EG) to cover 40 and 80% concentration range. To attain high osmolarity conditions, glycerol was replaced by EG because EG solutions are less viscous than those containing glycerol, improving sample handling and reproducibility. Glycerol was used at concentrations of 40% and 80% equivalent to 5.47 and 10.95 Osm, respectively. EG was used at concentrations of 40% and 80% corresponding to 7.17 and 14.34 Osm, respectively. Proteins mixes were analyzed in a Jasco J-715 CD spectropolarimeter (JASCO Analytical Instruments) in a 1 mm pathlength cell, using wavelengths from 190 to 260 nm. Temperature was maintained at 25 °C with a Peltier temperature-controlled cell holder (PTC-4235; JASCO). Three spectra were obtained for every mix, averaged, and smoothed to reduce noise. The spectropolarimeter was set to obtain readings of 5 s per nm. Data obtained was transformed to Mean Residue Weight ellipticity using Dichroweb Server^45^, using the CDSSTR algorithm and the SP175t dataset.

### Freeze–thaw protection assay

Freeze–thaw assays were used in this work because these treatments create a condition where water availability is reduced, a consequence of the solid-state configuration assumed by the water molecules. Protection efficiency against freeze–thaw treatment of LEA proteins was evaluated according to Rendon-Luna, et al.^14^. A master mix (final volume 600 µL) was prepared containing 25 mM Tris–HCl pH 7.5, LDH (rabbit muscle, Sigma-Aldrich) and the protectant to be evaluated in monomer molar ratios as indicated. For normalization of the LDH activity, we considered as 100% of the activity the value obtained for the enzyme:protector mix without the freeze–thaw treatment. The possibility that the wild-type LEA4-5 and its derivatives could aggregate during this treatment was discarded by monitoring turbidity at 3.

### Statistical analysis

To determine statistically different means in the cryoprotection assays, a one-way ANOVA, followed by a Dunnett’s multiple comparison post hoc test (α = 0.05) was performed, considering many-to-one comparisons, where the mean cryoprotection of each mutant was compared to the mean of cryoprotection of the LEA4-5 native protein. For all experiments, we conducted at least three independent experimental reproductions of the assays/experiments, including three technical replicates.

### Supplementary Information

Supplementary Table S5.

Supplementary Information 1.

Supplementary Information 2.

## Data Availability

All data generated or analyzed during this study are included in this published article (and its Supplementary Information files). The large dataset used for the phylogenetic analysis is available in the LEA4_proteins repository of Open Science Framework (https://osf.io/qj7n8/?view_only=1f1cb35ae8d74905b177988d18b3453b).
