# Supplementary material for: Alternative conformations of a group 4 Late Embryogenesis Abundant protein associated to its in vitro protective activity
Source: Sci Rep. 2024 Feb 2;14:2770. doi: 10.1038/s41598-024-53295-7 (PMC10837141; doi:10.1038/s41598-024-53295-7)
Supplement: Supplementary file 2 — Supplementary Information 1. [file 41598_2024_53295_MOESM2_ESM.pdf]

Alternative conformations of a group 4 Late Embryogenesis Abundant protein associated to its *in vitro* protective activity.

David F. Rendón-Luna[1], Inti A. Arroyo-Mosso[1], Haydee De Luna-Valenciano[1§], Francisco Campos[1], Lorenzo Segovia[2], Gloria Saab-Rincón[2], Cesar L. Cuevas-Velazquez[3], José Luis Reyes[1], and Alejandra A. Covarrubias[1]*

*Corresponding author: Alejandra A. Covarrubias.
alejandra.covarrubias@ibt.unam.mx

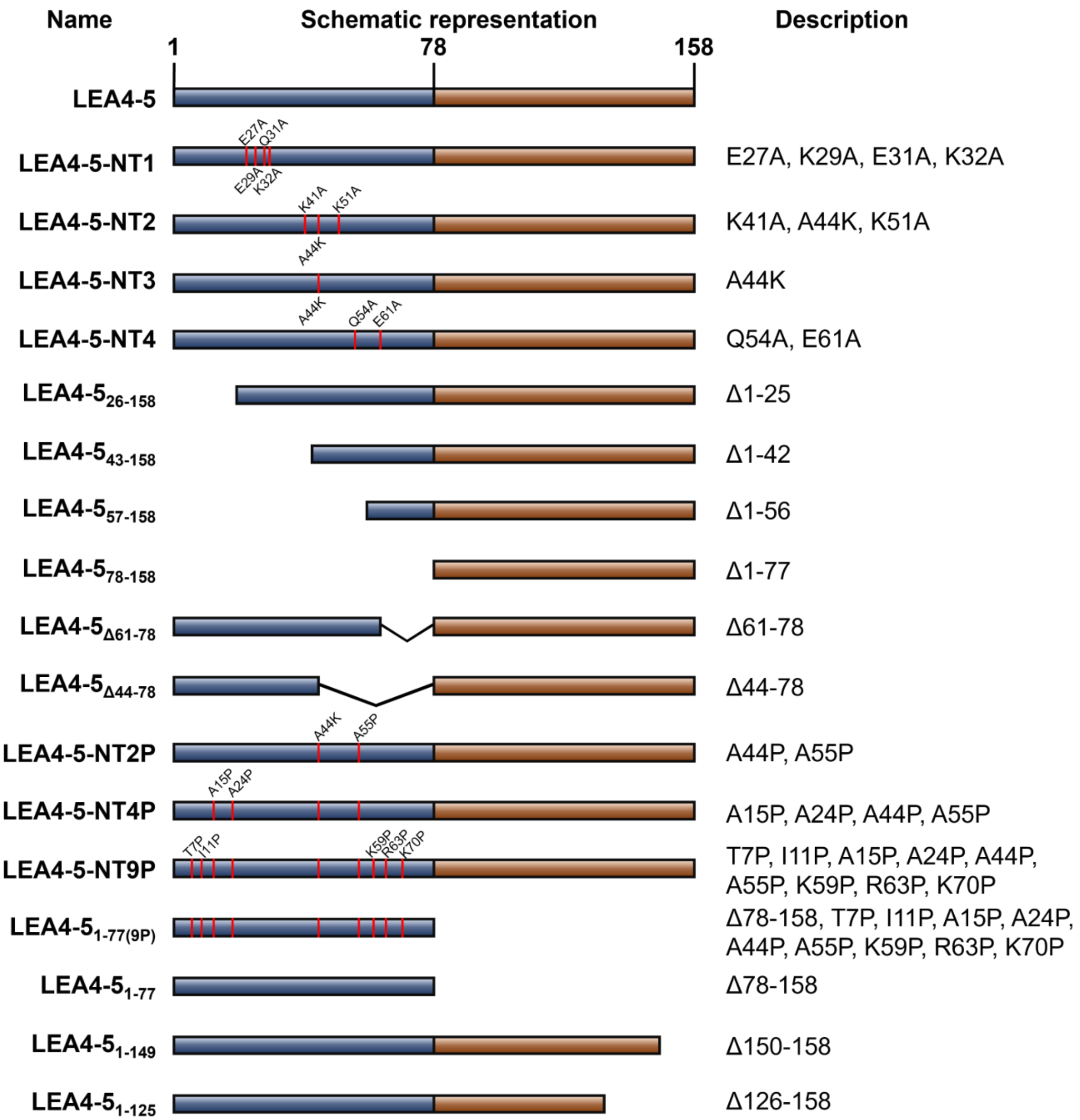

Supplementary Figure S1. Schematic description of wild-type LEA4-5 and derived mutant proteins analyzed in this study. Some of the physicochemical properties of the proteins in this figure are described in Supplementary Table S5.

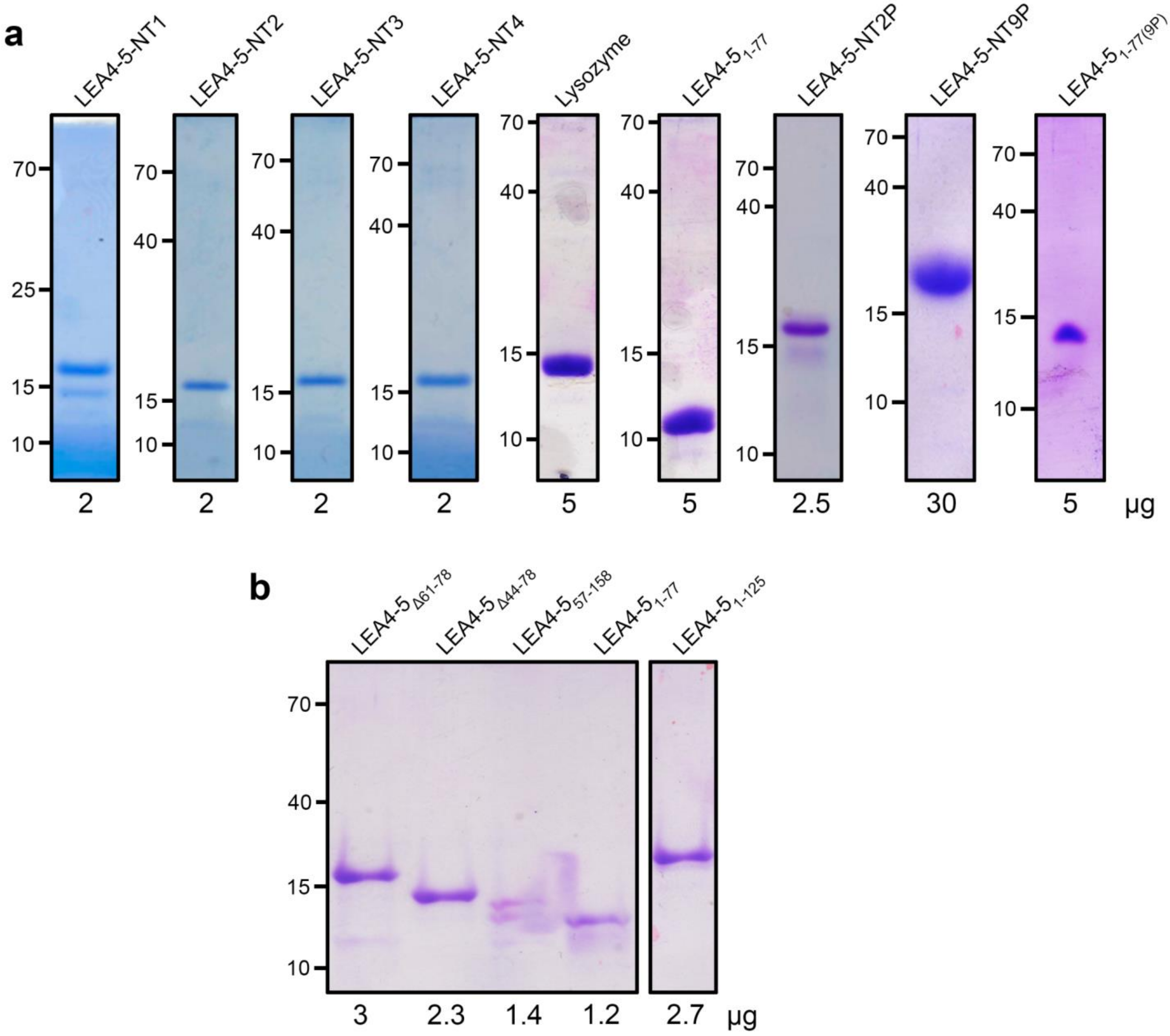

Supplementary Figure S2. SDS-Polyacrylamide gel electrophoresis separation of mutant proteins. The purity of the mutant proteins was verified by SDS-PAGE and Coomassie blue (G or R) staining. Proteins were quantified by the Qubit protocol (see Methods). The protein amount loaded in each lane is indicated at the bottom. The images of the purified protein samples that were separated in different gels are shown individually. The complete lane is shown in all cases. Numbers at the left of each lane correspond to the migration of the molecular weight markers in kDa (Pre-staining protein ladder, Thermo Fisher Scientific) for the respective gels.

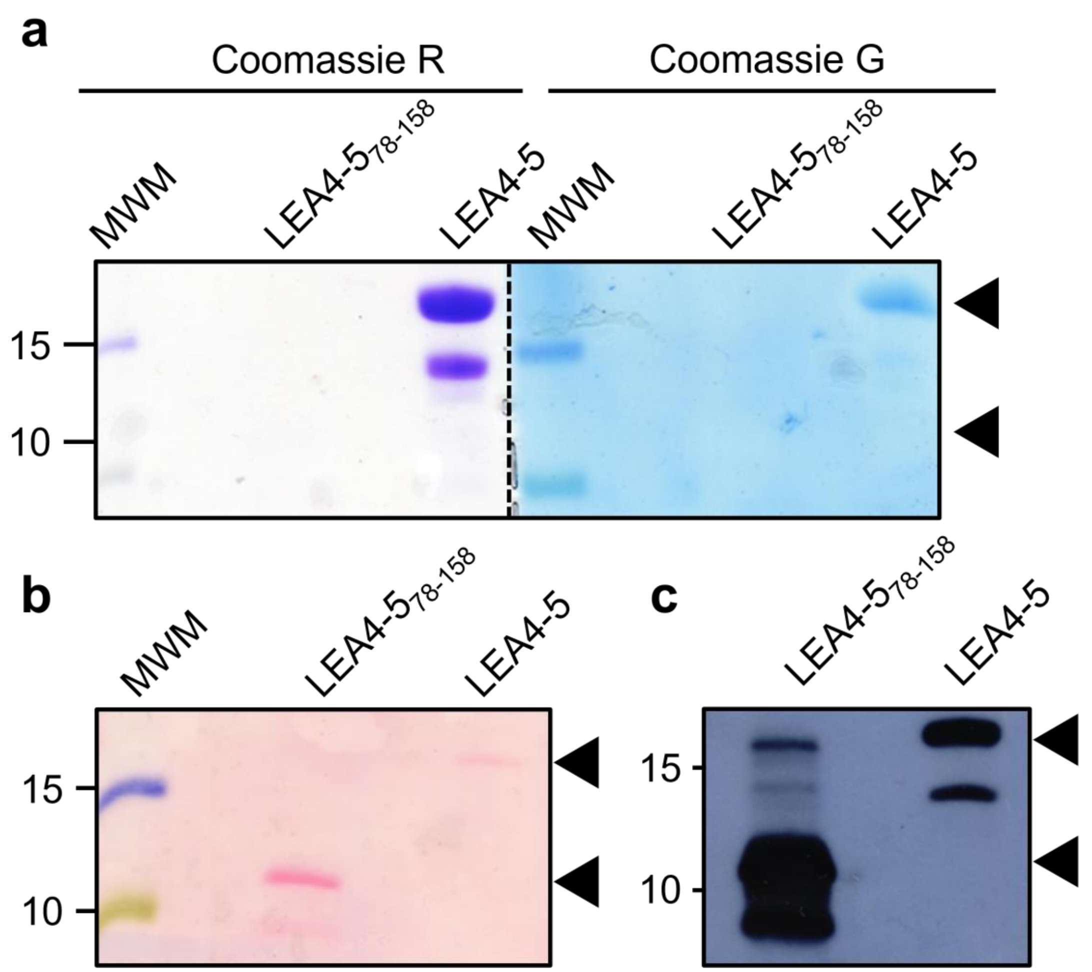

Supplementary Figure S3. Purity verification of LEA4-5$_{78\text{-}158}$ protein. A) Coomassie Blue R or Coomassie Blue G do not stain LEA4-5$_{78\text{-}158}$ mutant protein, conforming the LEA4-5 C-terminal region. This image shows the staining of the molecular weight markers and the LEA4-5 protein (5 µg) but not of LEA4-5$_{78\text{-}158}$ (5.5 µg). B) LEA4-5$_{78\text{-}158}$ was stained by Ponceau red upon transfer to a nitrocellulose membrane (pore size of 0.2 µm); a band corresponding to the LEA4-5 protein was also detected by this staining. Arrowheads indicate the location of the bands of these two proteins. C) Western blot experiments performed with a C-terminal-region-specific antibody (1:2,500) using anti-HRP (1:10,000) as secondary antibody. Black arrowheads point at the bands corresponding to LEA4-5 and LEA4-5$_{78\text{-}158}$ proteins migration. MWM: Molecular weight markers in kDa.

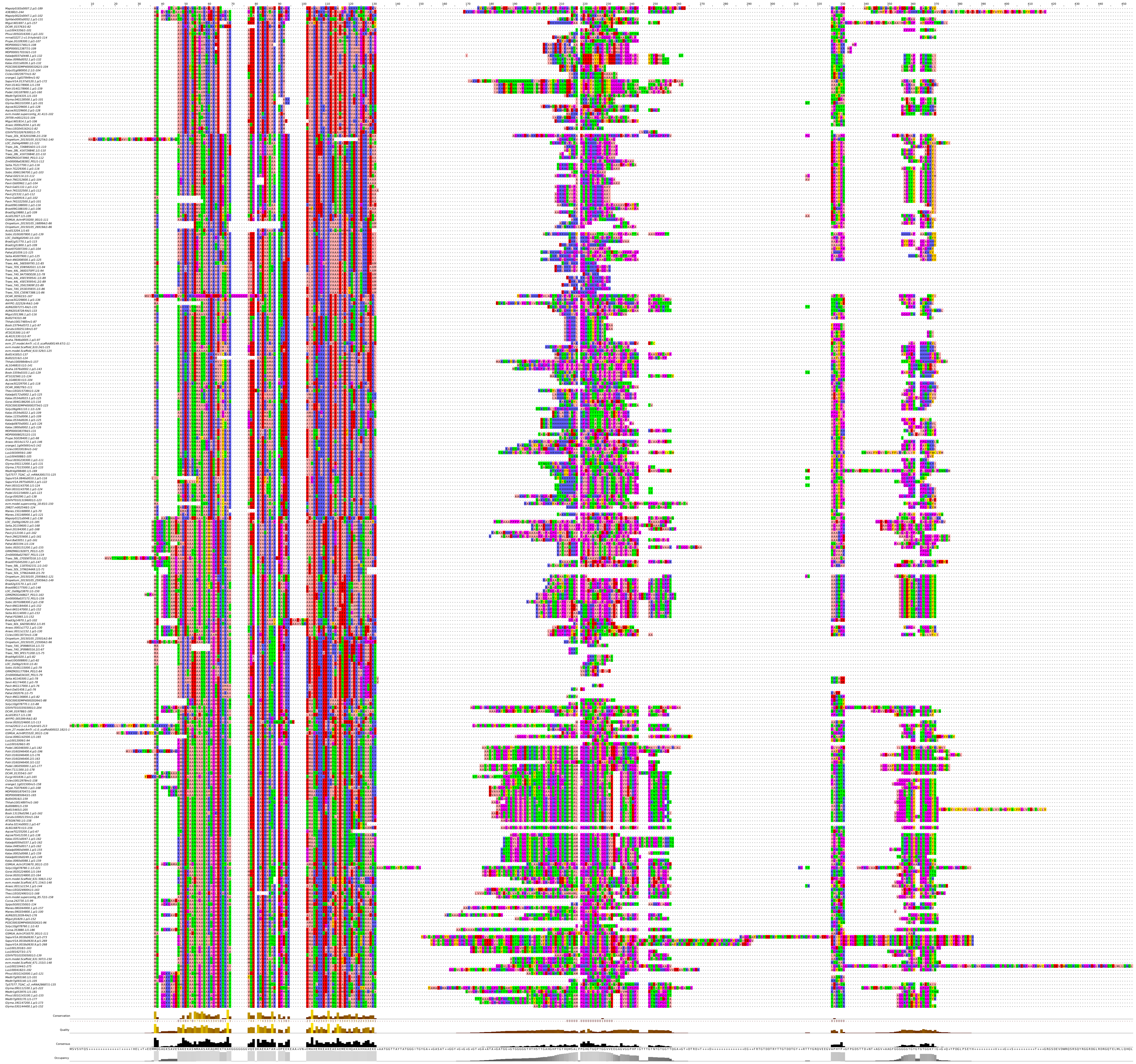

Supplementary Figure S4. Complete alignment of plant LEA4 proteins. This alignment was done with all proteins used in the phylogenetic analysis. Visualization, conservation, quality, and consensus sequence analysis were performed by Jalview software. Residues were colored as follows: red: negatively charged residues; blue: positively charged residues; green: polar uncharged residues; pink; residues with hydrophobic side chain; magenta: glycine and proline; orange: tyrosine and phenylalanine, and yellow: cysteine.

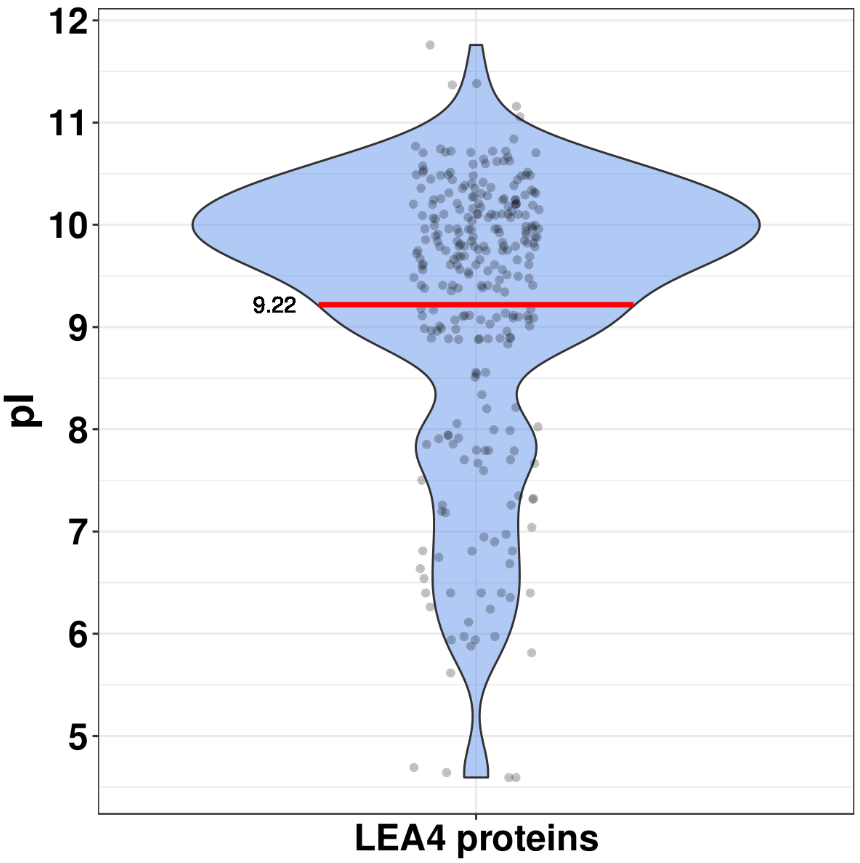

Supplementary Figure S5. Isoelectric point distribution of the LEA4 proteins using in the phylogenetic analysis. This analysis was performed using the Peptides package in the R programming language (Osorio, D., Rondón-Villarreal, P. & Torres, R. J. S. Peptides: a package for data mining of antimicrobial peptides. Small 12, 44-444 (2015). https://doi.org/10.32614/RJ-2015-001) . Each point corresponds to the pI value for each of the 275 LEA4 protein sequences. The red horizontal line represents the mean pI for this set of proteins.

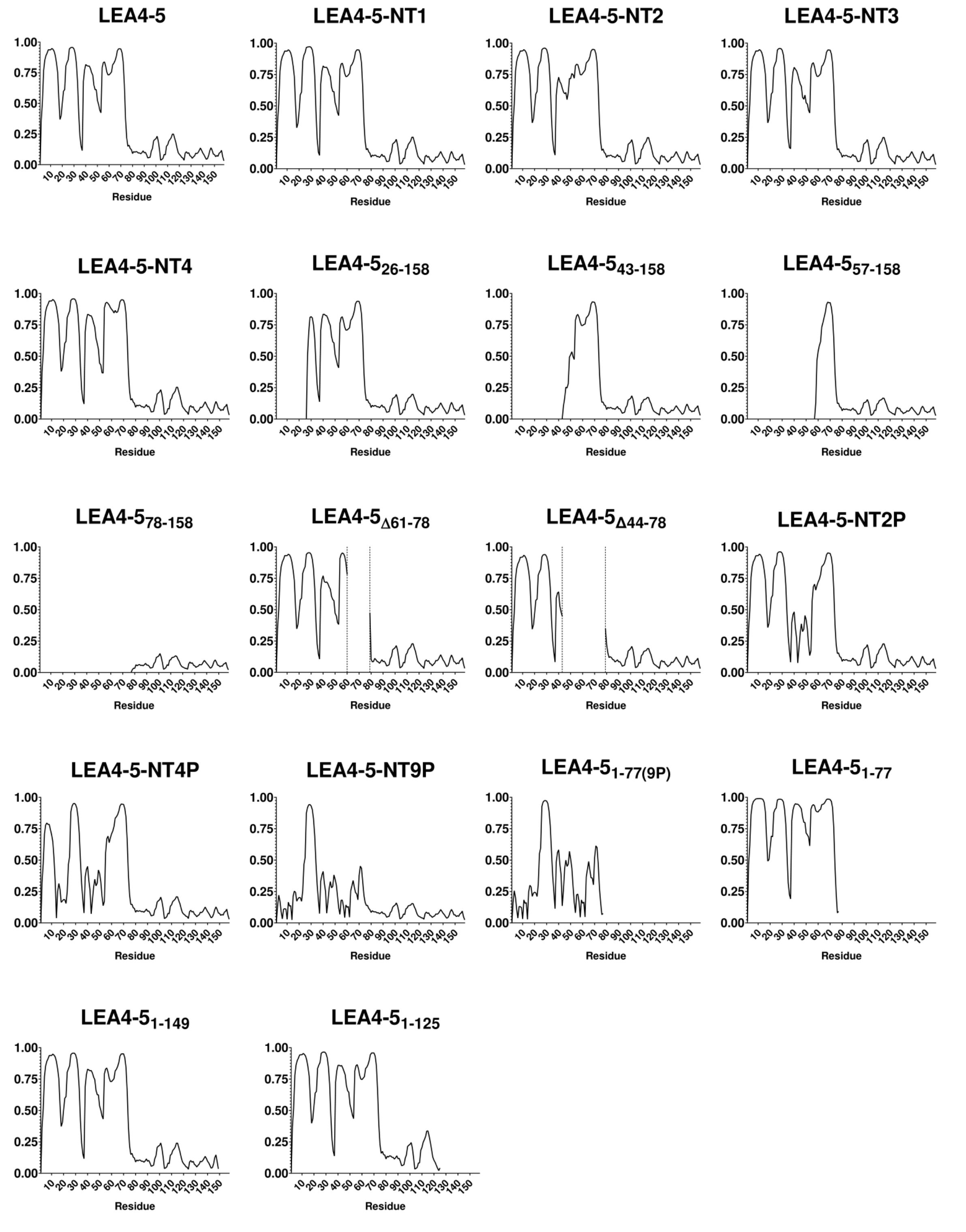

Supplementary Figure S6. Bioinformatic prediction of alpha-helix propensity using the FELLS algorithm. Y-axes show the alpha-helix propensity score assigned by this algorithm.

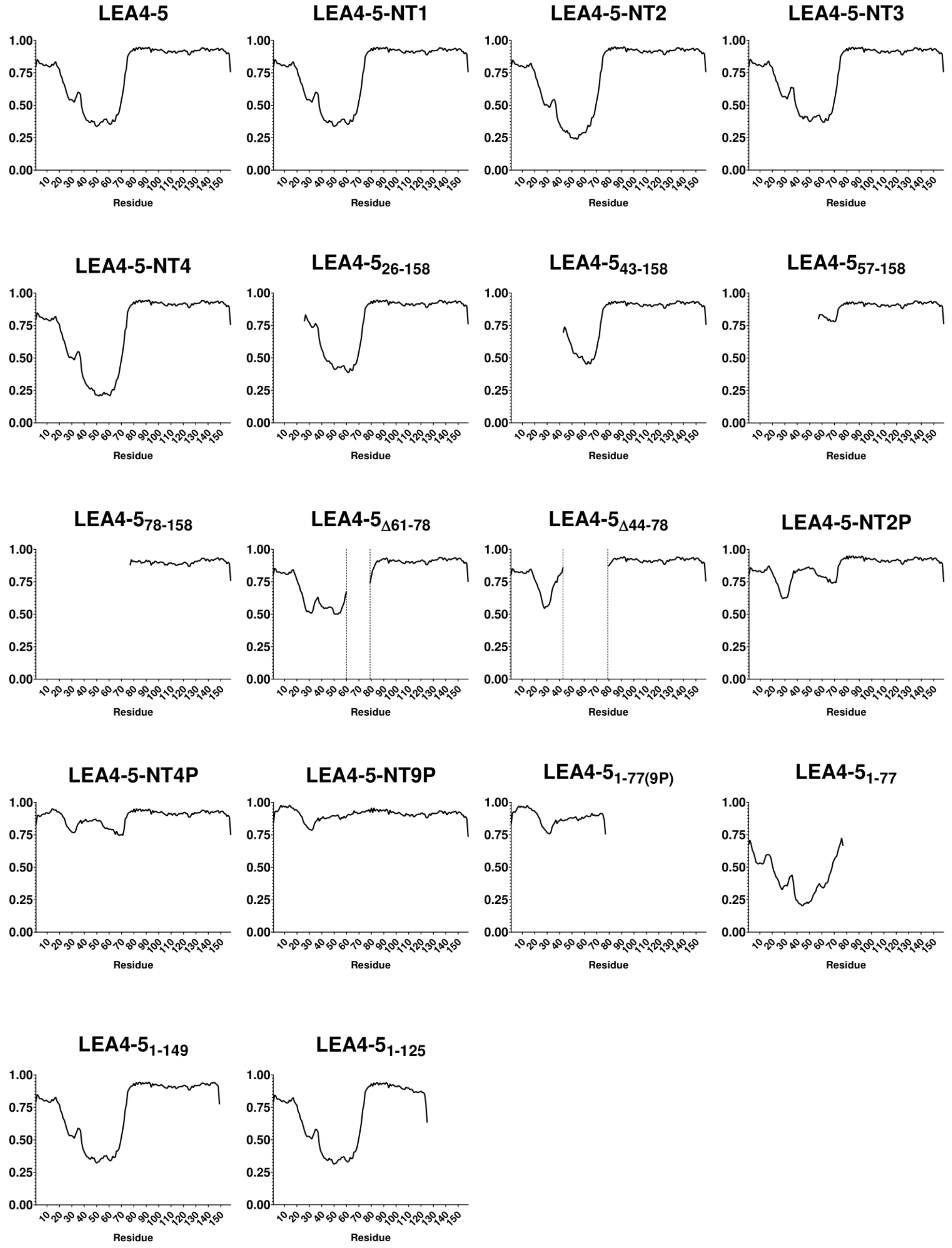

Supplementary Figure S7. Bioinformatic prediction of structural disorder using the Metapredict algorithm. Y-axes show the disorder propensity score assigned by this algorithm.

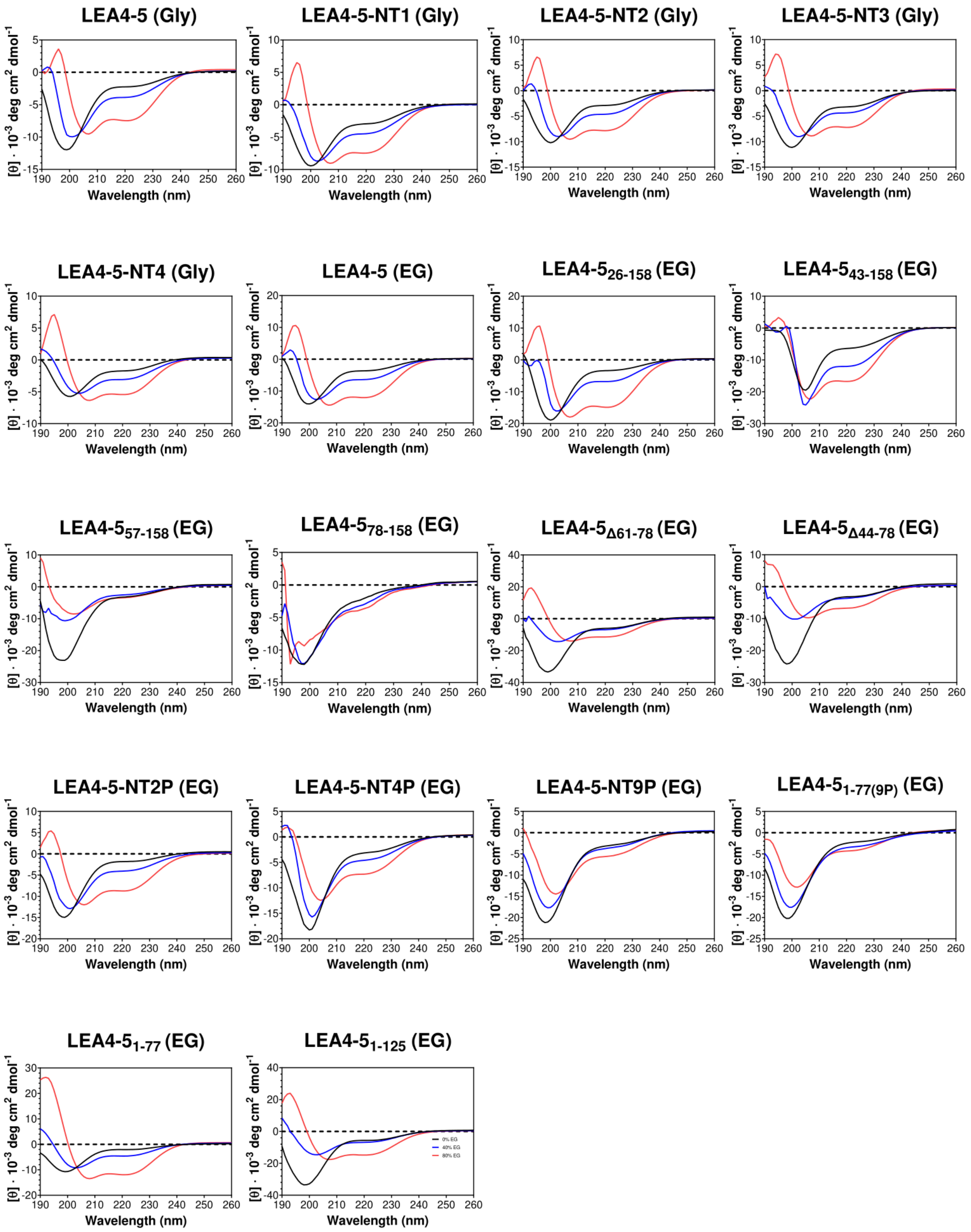

Supplementary Figure S8. Circular dichroism of the proteins analyzed in this study. Gly and EG refers to glycerol and ethylene glycol, respectively. Black, blue, and red spectra represent 0%, 40% and 80% concentrations of the water deficit inducer, respectively. [Φ] = Molar ellipticity.

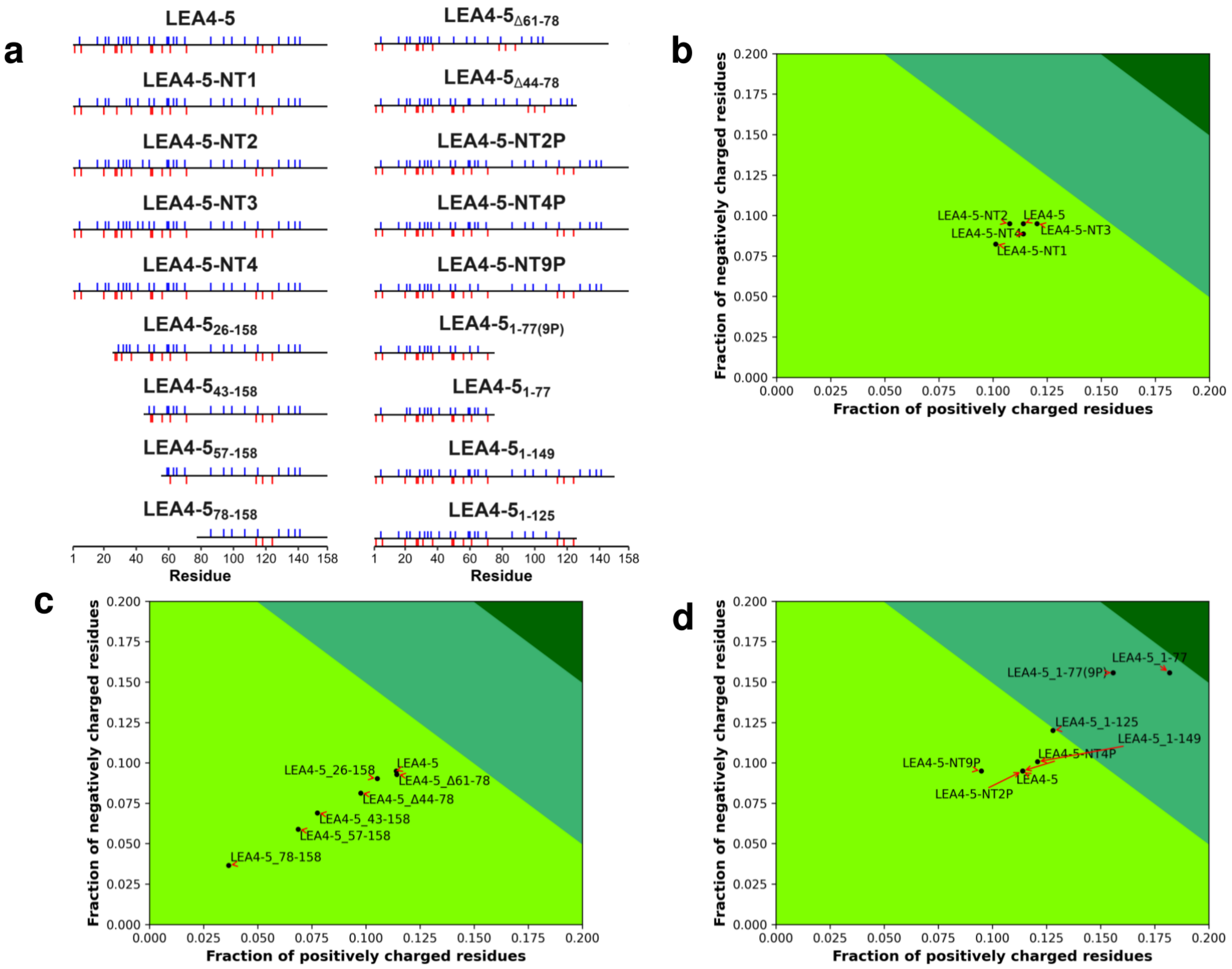

Supplementary Figure S9. Distribution of the charged residues in all the mutant proteins. A) Linear distribution of charged residues in the sequences of the different proteins characterized in this study. The position of the residues is indicated below the scheme. Blue: positively charged residues. Red: negatively charged residues. Distribution of the AtLEA4-5, wild-type, and mutant derivatives in the Das-Pappu diagram of state. This diagram offers an idea of the conformational behavior of the unstructured ensembles in disordered proteins [45]. B) LEA4-5, LEA4-5 NT1 - NT4. C) LEA4-5, LEA4-5$_{26-158}$, LEA4-5$_{43-158}$, LEA4-5$_{57-158}$, LEA4-5$_{77-158}$, LEA4-5$_{\Delta44-78}$, and LEA4-5$_{\Delta61-78}$. D) LEA4-5, LEA4-5$_{1-77}$, LEA4-5$_{1-77(9P)}$, LEA4-5-NT2P, LEA4-5-NT4P, and LEA4-5-NT9P. Bright green zone: weak polyampholytes and polyelectrolytes. Green: boundary region. Dark green: strong polyampholytes.

Supplementary Tables

Table S1. Sequences of oligonucleotides used to obtain the mutant proteins. Underlined and italic sequences correspond to restriction sites. Underlined and bold sequences correspond to modified codons.

Name	Sequence (5' → 3')
**TRC45F**	AAA *CCA TGG* AGT CGA TGA AAG AAA C
**TRC45R**	GCG *GTC GAC* CCG TTT ATC CAG TAT ATC C
**45F-27a32**	CAA AAC CAA AGC TAC CTT G**GC G**GA A**GC G**GC G**GC GGC G**AT GAA GAC ACG AGA CCC
**45R-27a32**	GGG TCT CGT GTC TTC AG**C GCC GC**C GC**C GC**T TC**C GC**C AAG GTA GCT TTG GTT TTG
**45F-41a51**	CGA GAC CCT GTT CAG **GCG** CAG ATG **AAA** ACA CAG GTT AAA GAA GAT **GCG** ATC AAT CAG GCT GAG
**45R-41a51**	CTC AGC CTG ATT GAT **CGC** ATC TTC **TTT** AAC CTG TGT TTT CAT CTG **CGC** CTG AAC AGG GTC TCG
**45F-A44K**	CCT GTT CAG AAA CAG ATG **AAA** ACA CAG GTT AAA GAA GAT AAG
**45R-A44K**	CTT ATC TTC TTT AAC CTG TGT **TTT** CAT CTG TTT CTG AAC AGG
**45F-54y61**	GAA GAT AAG ATC AAT **GCG** GCT GAG ATG CAG AAG AGA **GCG** ACG CGT CAG CAC AAC
**45R-54y61**	GTT GTG CTG ACG CGT **CGC** TCT CTT CTG CAT CTC AGC **CGC** ATT GAT CTT ATC TTC
**MtL45P12Fw**	CGA ATA TTG CAG CTT CT**C CA**A AAT CTG GCA TGG ACA AAA CCA AA**C CT**A CCT TGG AGG AAA AG
**MtL45P12Rv**	CTT TTC CTC CAA GGT **AGG** TTT GGT TTT GTC CAT GCC AGA TTT **TGG** AGA AGC TGC AAT ATT CG
**MtL45P34Fw**	CTG TTC AGA AAC AGA TG**C CT**A CAC AGG TTA AAG AAG ATA AGA TCA ATC AA**C CT**G AGA TGC AGA AGA GAG
**MtL45P34Rv**	CTC TCT TCT GCA TCT C**AG G**TT GAT TGA TCT TAT CTT CTT TAA CCT GTG T**AG G**CA TCT GTT TCT GAA CAG
**M33LAPNonu-F**	AAA CCA TGG AGT CGA TGA AAG AAC CAG CTT CGA ATC CTG CAG CTT CTC C
**M33LAPNonu-R**	GGC GTC GAC TCA GGT TCC GGC TCC AGC CGC TTC TGG CAT GGC CGC GTT GTG CTG AGG CGT TTC TCT TGG CTG CAT CTC
**M33LAPNonu-R-no-ter**	GGC GTC GAC ACC GGT TCC GGC TCC AGC CGC TTC TGG CAT GGC CGC GTT GTG CTG AGG CGT TTC TCT TGG CTG CAT CTC
**SD26.Fw**	TA*C CAT G*GA GGA AAA GGC GGA G
**M430.Fw**	AAA *CCA TGG* CTA CAC AGG TTA AAG
**LEA4M57.Fw**	AAA *CCA TGG* AGA AGA GAG AAA CGC G
**H61RV**	GGT GGT GTC GAC TCA TTC TCT CTT CTG CAT CTC AG
**Moxo1.Rv**	GCG *GTC GAC* TTA CCC GGT GGC ACC ACC
**Moxo2.Rv**	GCG *GTC GAC* TTA CGG GTC GGT CAC AGC CG
**FwGap**	GAT GCA GAA GAG ATT AGG TTT GGG GAC GGC C
**GapRv**	GCT GAG ATG CAG AAG AGA TTA GGT TTG GGG
**F43Gap**	CAG AAA CAG ATG TTA GGT TTG GGG ACG GCC
**Gap43R**	CCC TGT TCA GAA ACA GAT GTT AGG TTT GGG G

**TYB11NonuF**	GGT GGT GGT T*GC TCT TC*C AAC ATG GAG TCG ATG AAA GAA CCA GC
**TYB11NonuR**	GGT GGT GGT *CCC GGG* TCA GGT TCC GGC TCC AGC CGC TTC TGG C
**TRC4RC-F**	AAA CCA TGG CCG GTT TAG GTT TGG GGA C
**CLEA4H-R**	TTA TCC GGC TCC AGC CGC TCC TTT CAT GGC
**pTRC99a_R**	GCG TTC TGA TTT AAT CTG TAT CAG G

Table S2. Pairs of primers used to generate the gene fragments needed for the overlap PCR.

**Mutant protein**	**Primer for segment A**		**Primer for segment B**	
	Forward	Reverse	Forward	Reverse
**LEA4-5-NT1**	TRC45F	45R-27a32	45F-27a32	TRC45R
**LEA4-5-NT2**	TRC45F	45R-41a51	45F-41a51	TRC45R
**LEA4-5-NT3**	TRC45F	45R-A44K	45F-A44K	TRC45R
**LEA4-5-NT4**	TRC45F	45R-54y61	45F-54y61	TRC45R
**LEA4-5-NT2P**	TRC45F	MtL45P34Rv	MtL45P34Fw	TRC45R
**LEA4-5-NT4P** [a]	TRC45F	MtL45P12Rv	MtL45P12Fw	TRC45R
**LEA4-5-NT9P** [b]	TRC45F	M33LAPNonu-R-no-ter	M33LAPNonu-F-no-ter	TRC45R
**LEA4-5$_{\Delta 61\text{-}78}$**	TRC45F	GapRv	FwGap	TRC45R
**LEA4-5$_{\Delta 44\text{-}78}$**	TRC45F	Gap43R	F43Gap	TRC45R

[a] The template for this mutant was the LEA4-5-NT2P mutant gene.
[b] The template for this mutant was the LEA4-5-NT4P mutant gene.

Table S3. Pairs of primers used to generate the truncated amplicons by end-point PCR.

Mutant protein	Forward primer	Reverse primer
**LEA4-5$_{26\text{-}158}$**	SD26.Fw	TRC45R
**LEA4-5$_{43\text{-}158}$**	M430.Fw	TRC45R
**LEA4-5$_{57\text{-}158}$**	LEA4M57.Fw	TRC45R
**LEA4-5$_{78\text{-}158}$**	TRC4RC-F	TRC45R
**LEA4-5$_{1\text{-}149}$**	TRC45F	Moxo1.Rv
**LEA4-5$_{1\text{-}125}$**	TRC45F	Moxo2.Rv
**LEA4-5$_{1\text{-}77}$**	TRC45F	CLEA4H-R
**LEA4-5$_{1\text{-}77(9P)}$** [a]	M33LAPNonu-F<br>TYB11NonuF	M33LAPNonu-R<br>TYB11NonuR

[a] The template for this mutant was the LEA4-5-NT9P mutant gene. This mutant was obtained in two rounds of PCRs. First, using the first pair of primers, we replaced the codon 78 (GGT) for a stop codon (TGA). Then, we use the second pair of primers to add restriction sites to clone the amplicon into pTYB11 expression vector.

Table S4. Structural proportion of the different proteins in aqueous solution or 80% water deficit inducer. The data was obtained using the Dichroweb platform.

Protein	Helix	Strands	Turns	Disordered
LEA4-5	0.03	0.39	0.14	0.43
LEA4-5 80% Gly	0.32	0.16	0.14	0.38
LEA4-5-NT1	0.04	0.36	0.14	0.44
LEA4-5-NT180% Gly	0.33	0.16	0.13	0.38
LEA4-5-NT2	0.04	0.36	0.14	0.44
LEA4-5-NT2 80% Gly	0.33	0.15	0.14	0.38
LEA4-5-NT3	0.04	0.35	0.15	0.44
LEA4-5-NT380% Gly	0.24	0.22	0.14	0.39
LEA4-5-NT4	0.03	0.38	0.12	0.44
LEA4-5-NT4 80% Gly	0.2	0.28	0.13	0.38
LEA4-5	0.05	0.34	0.15	0.45
LEA4-5 80% EG	0.5	0.11	0.13	0.28
LEA4-$5_{26\text{-}158}$	0.04	0.34	0.15	0.46
LEA4-$5_{26\text{-}158}$ 80% EG	0.57	0.11	0.13	0.19
LEA4-$5_{43\text{-}158}$	0.46	0.12	0.1	0.32
LEA4-$5_{43\text{-}158}$ 80% EG	0.6	0.08	0.14	0.18
LEA4-$5_{57\text{-}158}$	0.03	0.34	0.14	0.48
LEA4-$5_{57\text{-}158}$ 80% EG	0.03	0.41	0.1	0.43
LEA4-$5_{\Delta 61\text{-}78}$	0.08	0.27	0.15	0.5
LEA4-$5_{\Delta 61\text{-}78}$ 80% EG	0.39	0.13	0.15	0.33
LEA4-$5_{\Delta 44\text{-}78}$	0.05	0.32	0.15	0.48
LEA4-$5_{\Delta 44\text{-}78}$80% EG	0.19	0.28	0.14	0.39
LEA4-$5_{1\text{-}125}$	0.08	0.27	0.15	0.51
LEA4-$5_{1\text{-}125}$ 80% EG	0.46	0.08	0.13	0.32
LEA4-$5_{1\text{-}77}$	0.05	0.37	0.15	0.42
LEA4-$5_{1\text{-}77}$ 80% EG	0.37	0.17	0.13	0.33
LEA4-5-NT2P	0.03	0.37	0.14	0.43
LEA4-5-NT2P80% EG	0.36	0.13	0.15	0.36
LEA4-5-NT4P	0.05	0.34	0.14	0.45
LEA4-5-NT4P80% EG	0.23	0.19	0.16	0.41
LEA4-5-NT9P	0.06	0.31	0.15	0.48
LEA4-5-NT9P 80% EG	0.16	0.25	0.17	0.43
LEA4-$5_{1\text{-}77(9P)}$	0.05	0.31	0.15	0.48
LEA4-$5_{1\text{-}77(9P)}$ 80% EG	0.08	0.31	0.16	0.44
