# Supplementary material for: Alternative conformations of a group 4 Late Embryogenesis Abundant protein associated to its in vitro protective activity
Source: Sci Rep. 2024 Feb 2;14:2770. doi: 10.1038/s41598-024-53295-7 (PMC10837141; doi:10.1038/s41598-024-53295-7)
Supplement: Supplementary file 3 — Supplementary Information 2. [file 41598_2024_53295_MOESM3_ESM.pdf]

Images with complete gels, membranes and films

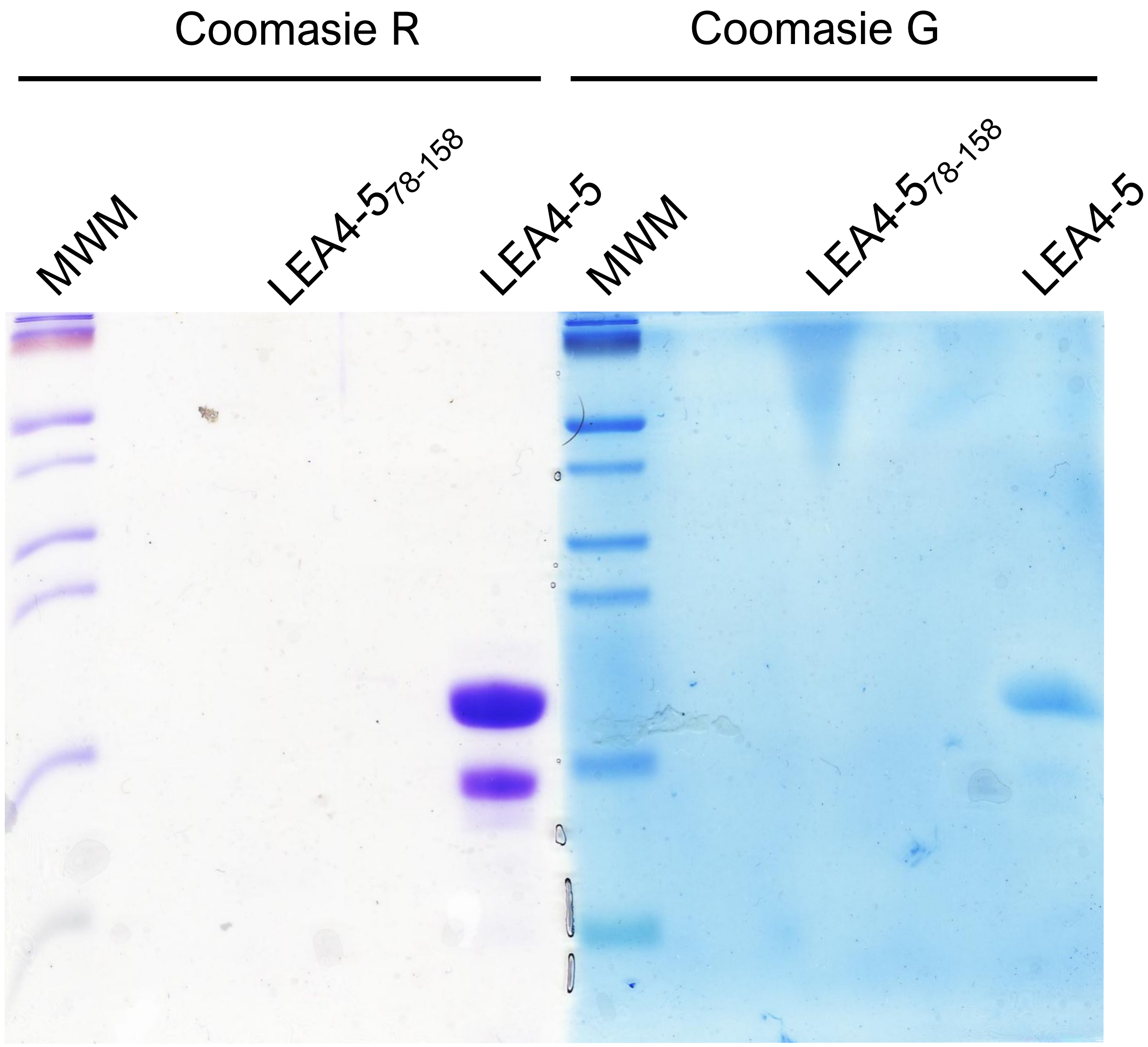

This is the complete image of the the gels shown in Supplementary Fig. S3a, which corresponds to one gel divided in two parts, one that was stained with Coomassie R and the other with Coomassie G. This gel loaded with two protein ladders and the exact same quantities of LEA4-5$_{78-158}$ and LEA4-5.

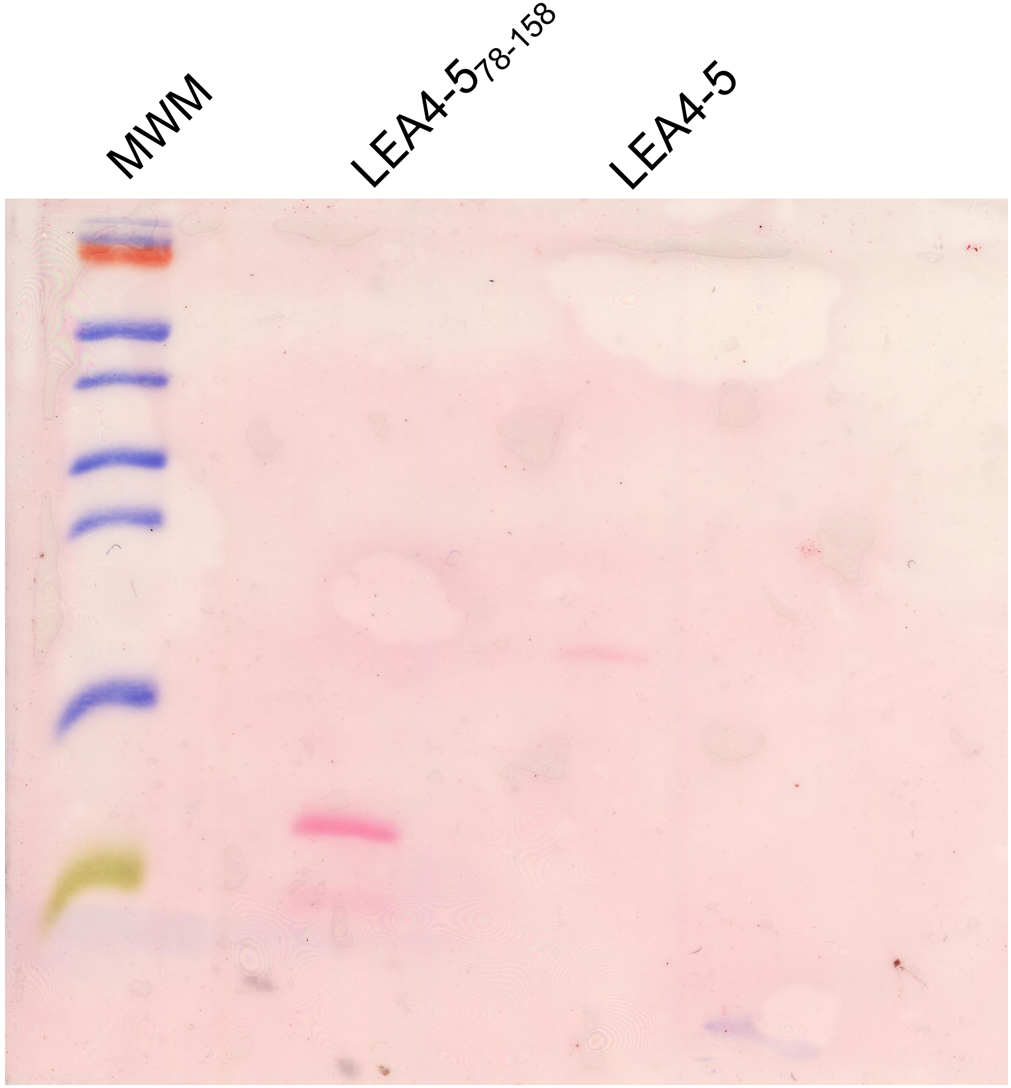

Image showing the complete Ponceau stained nitrocellulose membrane, presented in Supplementary Fig. S3b, into which MWMs and LEA4-5$_{78-158}$ protein were transferred after separation in SDS-PAGE, where the amount of protein loaded was the same as in the left stained gel-

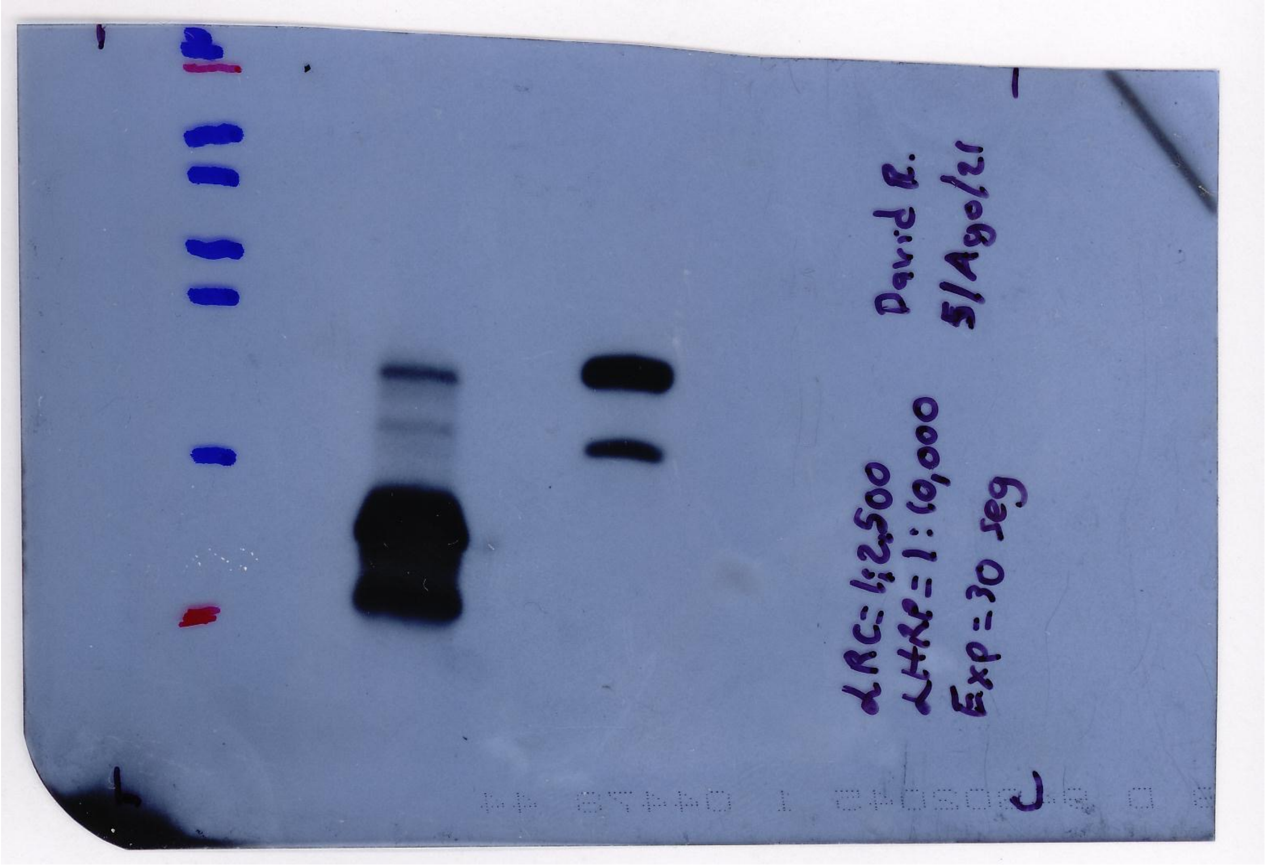

Image showing the compete film presented in Supplementary Fig. S3c, corresponding to a Western blot where Anti-LEA4-5$_{78\text{-}158}$ (1:2500) and Anti-HRP (1:10,000) were used. This film (Kodak Blue X-ray) was exposed for 30 seconds.

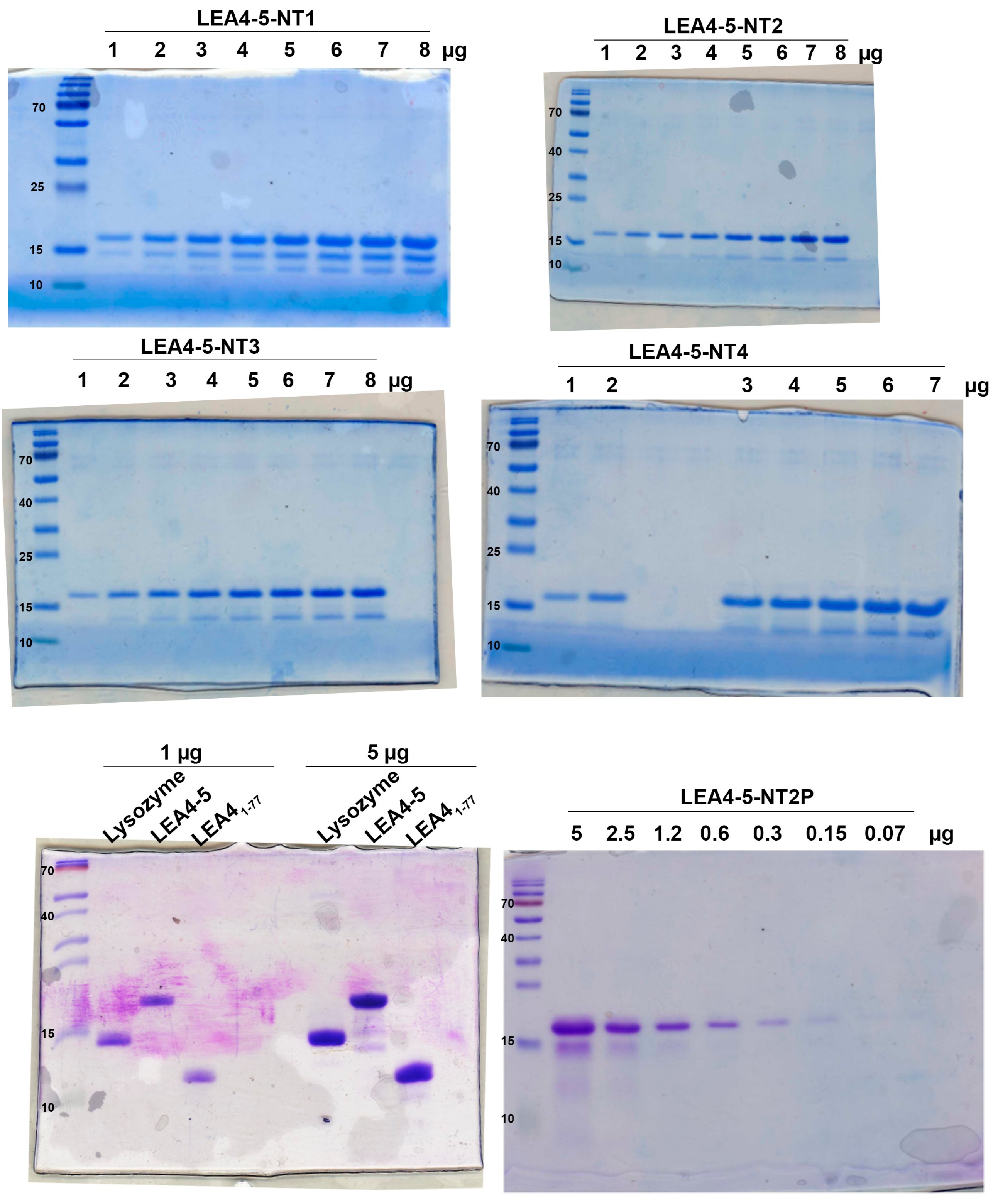

These complete gels correspond to the lanes shown in the panel (a) of Supplementary Fig. S2. The labels at the top of each gel described the samples separated in each of them.

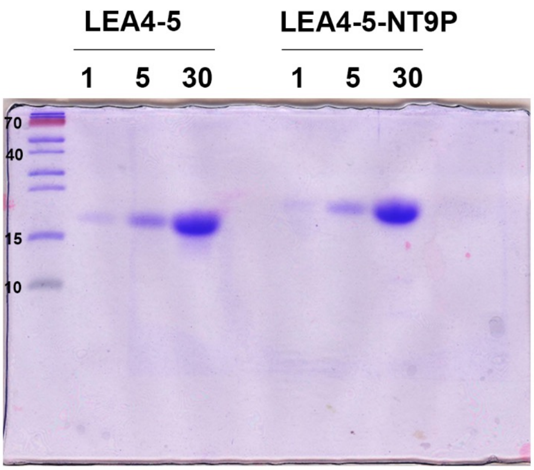

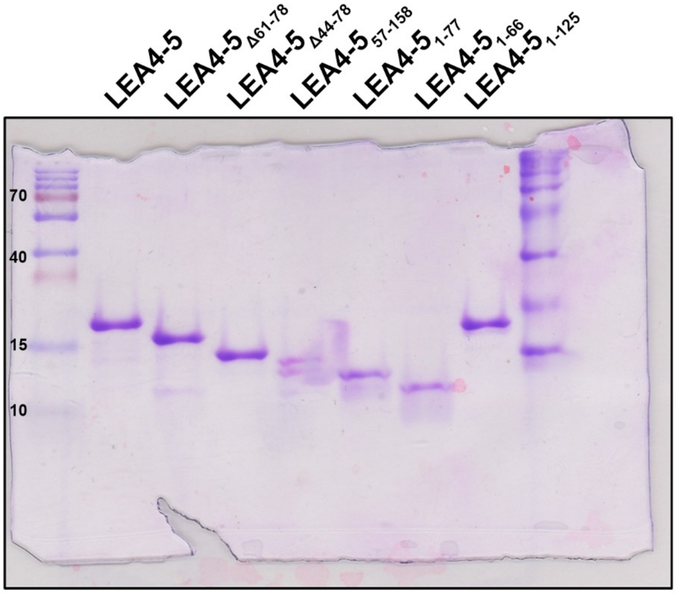

These complete gels correspond to the lanes shown in the panel (b) of Supplementary Fig. S2. The labels at the top of each gel described the samples separated in each of them.
